# Exploring Behavioral Interventions to Enhance Adherence to Multiple Micronutrient Supplementation Among Pregnant Women in Cambodia: A Mixed-Methods Study

**DOI:** 10.3390/nu18040583

**Published:** 2026-02-10

**Authors:** Cassandra Sauer, Meng Sokchea, Sambo Sreang, Hou Kroeun, Vannary Hun, Aman Sen Gupta, Kim Rattana, Mary Chea, Mai-Anh Hoang

**Affiliations:** 1 Food, Nutrition & Health, The University of British Columbia, Vancouver, BC V6T 1Z4, Canada; csauer@student.ubc.ca; 2 Helen Keller Intl, Phnom Penh, #40, Street 348, Sangkat Toul Svay Prey 1, Khan Boeung Keng Kang, Phnom Penh 120106, Cambodia; 3 Helen Keller Intl, Bhyuti Marg, Lalitpur 44700, Nepal; 4 National Maternal and Child Health Center, Ministry of Health, Phnom Penh 12202, Cambodia; 5 National Nutrition Program, Ministry of Health, Phnom Penh 12000, Cambodia; 6 Helen Keller Intl, One Dag Hammarskjold Plaza Floor, 2nd Ave, New York, NY 10017, USA

**Keywords:** multiple micronutrient supplements, pregnancy, adherence, implementation science, Cambodia, behavior change, COM-B model

## Abstract

Background/Objectives: Multiple micronutrient supplements (MMS) are more effective in improving maternal and birth outcomes compared to iron and folic acid (IFA) supplementation during pregnancy. High adherence to MMS is critical to achieving all the anticipated health benefits. Therefore, to ensure successful program implementation of transitioning to MMS in Cambodia, implementation research has been prioritized. The objective of this study was to assess the relevance, acceptability, utility and short-term, exploratory adherence outcomes of three behavioral interventions designed to support adherence to daily MMS intake and to promote healthy pregnancy practices. Methods: This mixed-methods study, conducted in four provinces in Cambodia, involved 12 focus group discussions (FGD) with 36 pregnant women who were exposed to three distinct behavioral interventions (family support, a tracking calendar, and educational videos). FGDs were held after three weeks of exposure to each intervention. Data was analyzed using a COM-B model, with inductive codes added to capture emerging themes. Results: Family support emerged as the most preferred and perceived as the most effective intervention, with participants consistently valuing the presence, encouragement, and practical assistance offered by husbands and other close family members. The second most preferred were educational videos, which were perceived to effectively convey key messages in an engaging, relatable way, overcoming literacy barriers. Finally, the calendar supported habit formation and appealed to first-time mothers but posed challenges for low-literacy users. Across interventions, findings reflect participants’ perceived effectiveness and preferences rather than comparative efficacy. Conclusions: This study provided insights into Cambodian women’s preferences and experiences with three distinct interventions designed to improve adherence over a short-term, 3-week exposure period per intervention. An integrated, multilevel approach that combines family-focused, educational, and system-level strategies is recommended for further piloting and scale-up, with longer-term studies needed to assess comparative efficacy for adherence and health outcomes.

## 1. Introduction

Micronutrient supplements are critical during pregnancy to meet the increased nutritional needs of pregnant women and to reduce the risk of adverse pregnancy and birth outcomes [1]. However, inconsistent intake of supplements is a barrier to receiving the full benefit for both the mother and infant. Micronutrient deficiencies can have both immediate and long-term adverse effects, including increased risks of maternal morbidity and mortality, pregnancy loss, congenital anomalies, impaired fetal growth and stunting, suboptimal neurocognitive development, and potentially higher cardiometabolic risk later in life [2,3,4].

Iron folic acid (IFA) supplementation has been the standard of care for pregnant women in Cambodia in accordance with the World Health Organization (WHO) guidelines [1,5]. In 2020, however, the WHO updated its recommendation, advising consideration of the United Nations International Multiple Micronutrient Antenatal Preparation (UNIMMAP) formulation of multiple micronutrient supplements (MMS). This shift has led to the initiation of implementation research on MMS in Cambodia and other countries to assess its effectiveness and feasibility [6,7]. In Cambodia, the move towards adopting MMS using implementation science to address key questions is strongly supported by government stakeholders and research is already underway [8,9].

High adherence to MMS is critical to achieving all the anticipated health benefits. Recent studies, including Smith (2025), demonstrate that only women with high adherence (≥90% of recommended doses) to MMS achieve significant improvements in birthweight, a reduced risk of low birthweight, and reductions in maternal anemia and stillbirth risk [10]. Those with low or inconsistent adherence experience fewer or no advantages over IFA supplementation. Thus, optimizing adherence must be central in MMS implementation efforts. 

While supply-side issues such as procurement, distribution, and health system readiness are often emphasized in discussions around supplementation programs, strong evidence suggests that behavioral, social, and contextual factors play a central role in influencing adherence. More specifically, behavioral change interventions are crucial and have been shown to yield significant improvements in maternal and infant outcomes [11]. There is evidence on interventions to increase supplement adherence, however, to be effectively implemented they need to be adapted to meet the specific context [12]. Therefore, research in Cambodia has focused on understanding the barriers and enablers that influence MMS adherence so that behavior change strategies can be designed and implemented to meet the needs of the local population. 

The emerging literature also shows that the ‘acceptability’ of supplementation is multidimensional, extending far beyond sensory attributes such as taste, appearance, or smell [13]. There is emphasis that acceptability encompasses practical aspects (such as packaging and ease of use), sociocultural fit, perceived effectiveness, support from family and healthcare providers, and perceptions regarding convenience and trust. For interventions to translate into sustained use, all these dimensions must be addressed within local implementation 

In 2023, a Helen Keller-led research team conducted in-depth interviews with 36 pregnant women in Kampong Thom province as part of a larger adherence and acceptability trial [8]. Participants were purposively selected to represent both low adherence (<80%) and high adherence (>95%) to MMS use. These interviews provided valuable insights into the barriers and enablers that influence MMS adherence. Common barriers to MMS adherence included forgetfulness and pregnancy-related symptoms such as nausea, constipation, and vomiting (unpublished data), while enablers included support from family and healthcare providers, as well as visual reminders at home (unpublished data). These findings highlight specific challenges that can be addressed to improve MMS adherence in Cambodia, as well as practical strategies already used by some Cambodian women to maintain consistent MMS intake during pregnancy. 

While the previous qualitative work in Cambodia identified key barriers and enablers of MMS adherence, it had not tested behavior change strategies with pregnant women or compared which approaches they are most willing and able to use in practice. This study employs a mixed-methods approach, similar to a Trial of Improved Practices (TIPs) and other user-tested approaches, which involve individuals trying specific practices and then providing recommendations on what is acceptable and feasible in daily life [14]. Such a study design helps generate comparative information on preferences and perceived effectiveness and is grounded in what people are willing to adopt. The objective of this study was to build off previous research by sequentially testing three distinct interventions—family support, a tracking calendar, and educational videos—and comparing their perceived acceptability, feasibility, and utility for supporting adherence to daily MMS intake and promoting healthy pregnancy practices.

As the Cambodian government prepares for a potential nationwide transition from IFA to MMS, implementing effective behavioral change and awareness-raising strategies to improve adherence is crucial for ensuring sustained use and improved health outcomes for pregnant women across the country. Understanding what drives or hinders women’s adherence at the individual and household level—including beliefs, habits, social support, and perceived benefits—is critical to designing interventions that are not only effective but also acceptable and sustainable in local contexts, considering all relevant dimensions of acceptability.

## 2. Materials and Methods

### 2.1. Study Design

This qualitative study was conducted from November 2024 to March 2025 across four provinces in Cambodia: Kampong Chhnang, Ratanakiri, Takeo and Kampot. A total of 36 pregnant women (*n* = 9 per province) were recruited from one health center in each of the four provinces. Participants were asked to take MMS throughout their pregnancy and engage with each of the three interventions for a period of three weeks. This 3-week exposure per intervention was chosen to align with a TIPs and related user testing approaches, which typically use brief (≈1–2 week) household trial periods to assess feasibility and acceptability, while allowing slightly more time for women to integrate each strategy into their daily routines [14,15]. Following each intervention, they were invited to participate in a focus group discussion (FGD). MMS tablets used in the study were donated by Kirk Humanitarian and followed the United Nations International Multiple Micronutrient Antenatal Preparation (UNIMMAP) MMS formulation and manufactured by Contract Pharmacal Corp., Hauppauge, NY 11788 USA [16]. MMS is included in the 2023 WHO Model List of Essential Medicines, proving safety and efficacy for public health interventions in the intended population [17]. Registration was not completed prospectively because the study team did not recognize that behavioral implementation research of this kind required trial registration, and prospective registration is not specified in the current NECHR SOPs [18]. The trial has since been retrospectively registered at ClinicalTrials.gov, NCT07388433.

### 2.2. Participants

Eligible participants were healthy pregnant individuals (18–45 years), within their first 14 weeks of a low-risk, singleton pregnancy (defined as the absence of any medical condition), and attending their first ANC visit at one of the participating health centers. Participants were required to reside in one of the four study provinces, have no plans to relocate within the next 4 months, be willing to take MMS as their prenatal supplement, and agree to participate in 3 FGDs. Individuals were excluded if they planned to relocate outside one of the four study provinces within four months or if their health care provider classified their pregnancy as high-risk.

### 2.3. Data Collection Tools

Semi-structured guides were developed for the FGDs to explore pregnant women’s preferences and experiences participating in three different interventions aimed at enhancing MMS adherence. The guides were systematically designed to explore participants’ perspectives on the acceptability, relevance, utility, and drawbacks of each intervention. Each FGD guide was initially drafted in English and subsequently translated into Khmer, the local language. The translated guides were pre-tested with pregnant women who were not enrolled in the study, and revisions were made based on participant feedback to improve clarity and cultural relevance. Full FGD guides are provided in the Supplementary Materials. All FGDs were audio-recorded using two Android tablets and note-taking was conducted using paper notepads to supplement the recording.

### 2.4. Recruitment and Training of the Research Team

A team of six data collectors, 5 females and 1 male, each of whom is a native Khmer speaker, university-trained and experienced in qualitative health research, was hired to collect study data. Their background and motivations were considered in both team selection and training processes. None of the researchers had any prior personal or professional relationships with the study’s participants or with the funding organizations. Before starting the study, all data collectors participated in an intensive 2-day training led by the core research team (M.S, M.A.H, C.S). This training included not only practical elements—such as review of the study’s protocol, standard operating procedures, ethical considerations, questionnaire administration, standardized facilitation, recording, note-taking, data management, and transcription—but also dedicated sessions on research reflexivity. These sessions highlighted strategies for maintaining neutrality, recognizing unconscious bias, and minimizing researcher influence on participant discussions. During all interactions, interviewers consciously withheld personal opinions about MMS or any intervention, ensuring that data collection reflected participants’ authentic views.

### 2.5. Recruitment and Data Collection

Recruitment at health centers began in November 2024, and enrollment continued until January 2025, with study completion in March 2025. One health center was selected in each of the four provinces based on the volume of pregnant women attending ANC visits. Healthcare providers trained on study procedures approached pregnant women during their first ANC visit for their interest in participating. FGDs were organized by province at the health center with a minimum of five participants required to proceed with each discussion lasting between 60–90 min and no repeat sessions conducted. All FGDs were conducted in Khmer and supported by a two-person team—one facilitator (M.S or S.S) and one dedicated notetaker. Only participants and trained researchers were present during data collection; no family members, health workers, or others were in the space at any time. Upon completing the session, each participant received a token of appreciation in the form of a sarong, towel, or water bottle.

### 2.6. Behavioral Intervention Trialed

We designed three interventions to pilot in the Cambodian context to determine their effectiveness in improving adherence to MMS.

#### 2.6.1. Family Support Intervention

The first intervention was family support, where each participant was asked to invite two family members—selected at the participant’s discretion based on who they considered most influential in their daily lives—to attend a single one-hour session at the health center. The session was conducted in Khmer and facilitated by trained midwives and research staff. The curriculum covered key ANC topics, the importance of daily MMS, and practical, actionable strategies for family members to support the participant’s adherence to MMS. The session was interactive, encouraging family members to ask questions and discuss potential challenges and solutions. At the end of the session, each family member received a summary handout outlining the main discussion points and specific actions they could take to support the pregnant participant’s health and supplement adherence. Fidelity to the intervention was assessed in the follow-up FGD with the enrolled pregnant women, held three weeks after the session, to capture their experiences and perceptions of family support in practice.

#### 2.6.2. Tracking Calendar Intervention

Following the FGD on the family support intervention, each participant received a culturally tailored tracking calendar to support adherence to MMS and promote healthy pregnancy behaviors. A social marketing consulting group was contracted to design and pretest the calendar with intended users, refining it iteratively based on feedback from the research team and pregnant women not enrolled in the main study across the four provinces. The calendar featured visual cues for marking daily MMS intake, serving as a visible record of adherence. It also included reminders for scheduled ANC visits and culturally appropriate icons promoting positive pregnancy practices such as balanced nutrition and rest, alongside guidance on behaviors to avoid, such as alcohol consumption and heavy lifting. Materials were developed in Khmer, using clear visuals and explanatory text to ensure accessibility for participants with varying literacy levels. Research staff provided a brief orientation for participants on using the calendar, demonstrating how to mark MMS intake, and interpret the icons. Three weeks later, follow-up FGD were held to explore participants’ experiences with the tool, including its perceived usefulness, challenges, and suggestions for improvement.

#### 2.6.3. Video Intervention

Finally, the video intervention was designed to leverage digital communication for promoting adherence to MMS among pregnant women. Over a three-week period, each participant received one motivational video per week via Telegram, a widely used messaging platform in Cambodia. The script for each video was developed collaboratively by the research team and reviewed by maternal health experts to ensure accuracy and clarity. The videos were in the Khmer language and featured midwives delivering counseling and encouragement specifically focused on daily MMS adherence. The videos incorporated practical tips, empathetic messaging, and visual cues to reinforce key messages. The content also addressed common pregnancy-related concerns and emphasized the importance of family support in maintaining consistent supplement intake. Prior to dissemination, the videos were pretested with a small group of pregnant women not participating in the main study. At the end of the intervention, participants were invited to a final FGD to discuss their experience with the videos including its perceived usefulness, challenges, and suggestions for improvement.

### 2.7. Evaluation and Data Collection

#### 2.7.1. Sociodemographic Data

Sociodemographic information—including age, ethnicity, religion, educational level, marital status, and gravida—was collected at participants’ homes in the local language by research staff using interviewer-administered questionnaires. These data were obtained through self-report, at the first household visit, to ensure accuracy and contextual understanding.

#### 2.7.2. Focus Group Discussions

After 3 weeks of exposure to each intervention, participants were invited to attend a FGD. The FGDs were designed to facilitate in-depth exploration of participants’ experiences with each intervention, including challenges and facilitators to MMS adherence, and provide feedback on the intervention content and delivery. At the final FGD, participants were asked to rank all three interventions in order of perceived effectiveness and explain their overall preferences. This sequential and qualitative approach allowed for a nuanced understanding of acceptability, relevance, and perceived effectiveness across interventions.

#### 2.7.3. Pill Counts

Adherence to MMS supplementation was objectively measured via tablet counts conducted at the end of each FGD. MMS tablets were poured into a clean, transparent plastic bag to ensure an accurate and hygienic counting process. Two separate counts were performed for each participant; if a discrepancy was identified, a third count was completed to verify the results. This process provided a reliable objective measure of supplement intake throughout the intervention periods.

#### 2.7.4. MMS Acceptability Survey

Acceptability data were captured through an oral, interviewer-administered quantitative questionnaire. Participants were asked to rate their agreement with statements about their perception of MMS—such as “I like the taste”, “The package gives me confidence that the supplements are from a quality manufacturer” and “The supplement is good for my baby’s health”—using a 5-point Likert scale (“strongly disagree”, “disagree”, “neutral”, “agree”, “strongly agree”). Participants were presented with corresponding emoticons and invited to select the emoticon that best represented their opinion for each statement, facilitating the ease of response. Upon enrollment, all participants were informed that adherence and acceptability data would be routinely collected during each FGD.

### 2.8. Data Management, Processing and Analysis

All FGDs were audio-recorded and supplemented with detailed field notes taken by trained note-takers. Immediately following each FGD, note-takers produced verbatim transcripts in Khmer. To ensure accuracy and completeness, transcripts were reviewed by two members of the research team (M.S and S.S). These original Khmer transcripts were then translated into English by bilingual translators with contextual familiarity and subsequently reviewed for accuracy and fidelity to the original meaning by M.S and C.S before analysis began. Due to logistical and resource constraints, transcripts were not returned to participants for review. However, synthesized findings were discussed with a subset of participants at the conclusion of FGDs to verify and contextualize emerging insights. No major discrepancies were identified.

Three trained coders on the research team analyzed the de-identified transcripts using NVivo software, version 14 (QSR International). We employed a content analysis approach that combines inductive and deductive coding. An initial codebook was developed deductively based on the study’s research objectives and the COM-B model (Capability, Opportunity, Motivation–Behavior) as the guiding theoretical framework [19]. As the analysis progressed, additional inductive codes were generated and discussed with the larger research team to ensure reflexivity, validate interpretation against the study context, and to capture emergent themes not covered by the initial framework. Team members brought diverse expertise in maternal health, behavioral science, and Cambodia specific program implementation, which enriched the analytical process. These collaborative discussions helped ensure transparency and analytical rigor, maintaining alignment between the data and the study’s theoretical and programmatic objectives.

To ensure intercoder reliability, one randomly selected transcript from each intervention was independently double coded by a second coder (C.S) using the same codebook. Coding agreement was assessed both quantitatively and qualitatively. If the inter-coder agreement fell below 90% or the Cohen’s kappa coefficient was below 0.6, differences were reviewed, and the team reconvened to discuss and refine code definitions. All discrepancies and divergent perspectives were resolved through discussion, and transcripts were re-coded as needed to maintain consistency and reliability across the dataset.

In analyzing the qualitative data, the COM-B framework was applied following an initial categorization of findings by their acceptability, relevance, and utilization. This approach allowed for the identification of key determinants influencing participant behavior related to MMS adherence. Coding and theme development were mapped directly onto the COM-B domains to ensure a robust, theory-driven analysis of the multifaceted factors affecting supplement adherence. The COM-B model guided interpretation, structuring analysis around three interlinked domains essential for behavior change: capability (knowledge and physical ability), opportunity (social and environmental enablers or barriers), and motivation (both reflective intentions and automatic reactions) [19]. After preliminary categorization by intervention acceptability, relevance, and utilization, emergent themes were mapped to relevant COM-B domains. This theory-informed process enabled systematic identification of the behavioral mechanisms contributing to MMS adherence across all interventions.

Quantitative data analysis used descriptive statistics to assess MMS adherence and intervention acceptability. Adherence was calculated by the number of tablets consumed divided by the number of tablets eligible to be consumed based on the participant’s first ANC visit to the date the tablets were counted. Acceptability was assessed using a structured quantitative questionnaire based on a published framework for the acceptability of healthcare interventions [20]. The six domains in the questionnaire include physical properties of the supplement, packaging, burden of taking, perceived effectiveness, opportunity cost, and self-efficacy. Participant responses were obtained approximately 90 days after the intervention. Agreement was calculated by totaling the responses in the “strongly agree” and “agree” categories, whereas disagreement was calculated by totaling the responses in the “strongly disagree” and “disagree” categories.

All data were securely stored on password-protected devices and cloud-based servers accessible only to designated research team members. Data confidentiality was maintained following ethical guidelines approved by the National Ethics Committee for Health Research in Cambodia. 

## 3. Results

### 3.1. Participant Characteristics

A total of 86 pregnant women were screened across four provinces (Table 1). Of these, 15 declined to participate, and 35 did not meet eligibility criteria. The remaining 36 pregnant women enrolled in the study received MMS throughout the study period and participated in the interventions. Thirty-one participants took part in the FGDs, as some participants who initially enrolled were unable to participate due to relocating for work or experiencing a miscarriage.

### 3.2. Adherence & Acceptability

Participants took MMS for an average of 79 days and achieved a median adherence of 97% (IQR: 92,100). Among participants who reported missing doses, the primary reasons included illness unrelated to MMS, being away from home without the supplements, and occasional forgetfulness. Acceptability of MMS was consistently high among participants. The highest levels of agreement were observed in the perceived effectiveness domain, highlighting participants’ strong belief in the importance of MMS for their own health, their infant’s well-being, and their overall pregnancy outcomes. Furthermore, participants reported a strong sense of self-efficacy, expressing confidence in their ability to take MMS daily and integrate the supplement into their routine. This high acceptability and self-efficacy underscore the relevance and feasibility of MMS use among the study population. 

### 3.3. Intervention Ranking

The following descriptive statistics summarize participants’ rankings of the three interventions by province (Table 2). During the final FGD, participants were asked to rank the three interventions—family support, tracking calendar, and educational video—according to which they preferred and perceived as most helpful for supporting daily MMS adherence and healthy pregnancy practices. Rankings were analyzed by province, and the combined results clearly identified family support as the most preferred and perceived as most helpful intervention, followed by the video intervention, with the tracking calendar ranking third. These rankings reflect participants perceived usefulness and preference rather than comparative effectiveness, and the qualitative design and small sample do not allow for formal efficacy comparisons between interventions.

### 3.4. Family Support Intervention

Data on the nature and frequency of family support were obtained through participant recall during FGDs. Support from husbands emerged as the most frequent and accepted form of familial involvement. Participants perceived that husbands’ physical proximity and regular interaction positioned them as key enablers of their supplement-taking routines and broader health behaviors. Analyzing family support through the COM-B framework reveals its multi-faceted role in enhancing women’s adherence to MMS in Cambodia (Table 3).

#### 3.4.1. Capability: Building Daily MMS Routines Through Family Support

Family support, particularly through regular reminders and assistance with routine tasks, significantly contributed to building women’s psychological and physical capability for adherence.

“When I had my family to remind me. I didn’t often forget, and I took supplement regularly… When they didn’t remind me, I took it by myself. Normally, I was busy I had morning sickness, I felt tired. So, I might take a rest and forget to take supplement.”

PW in Kampong Chhnang

Participants described how consistent encouragement from family members helped establish MMS intake as part of their daily routine. Over time, adherence became habitual and required less cognitive effort:

“I remembered to take supplements in the evening because they reminded me ever since the early of the month; they always reminded me to take supplements. Now, when I could remember to take supplements, they didn’t often remind me.”

PW in Kampot

In addition to prompting regular supplement-taking behavior, family members played an active role in knowledge sharing. By becoming informed about the importance of MMS and healthy pregnancy practices in the family support session, family members were better positioned to reinforce accurate health messages and support behavior change. This knowledge transmission further empowered women to make informed health decisions and maintain adherence.

“Before, they didn’t often remind and care about me. After coming to the session, they often reminded and explained to me about this or that.”

PW in Ratanakiri

“When he came to the session, he understood a lot. He didn’t let me have alcohol or coffee because it weakened the baby health.”

PW in Kampong Chhnang

#### 3.4.2. Opportunity: Household Support and Shared Responsibilities

The presence and engagement of husbands and other household members created both physical and social opportunities that enabled consistent supplement use. Families were often aware of women’s daily routines and could provide timely, personalized support. This included not only verbal reminders but also tangible assistance such as preparing meals, managing household chores, and reducing the physical workload of pregnant women:

“Before she came, when I got supplements from the health center, he didn’t care much. After she came to teach him, he started to help prepare meals and supplements for me or he called to ask/check me. Before, he was not like that.”

PW in Kampot

“For me, my husband bought vegetables, fruits, milk and kept them in the cabinet. He helped me a lot. When I was feeling unwell due to morning sickness, he helped to do laundry.”

PW in Kampot

Such support fostered a socially enabling environment in which women felt cared for and understood.

“When he was given the session, he understood that I took supplements. So, I was able to take supplements more regularly compared to before. Before he didn’t know, and I just took supplements on my own. Whether or not anyone knew it, I still took supplements. Now, after he was given the session, he knew that I took supplement and it made me feel happy. So, I felt happy when I had more people to know and reminded me to take supplements every time.”

PW in Kampot

The intervention’s relevance was heightened by the physical proximity of family members, especially husbands, who were ideally situated to integrate health-promoting behaviors into daily life. However, in households where family members were physically absent or emotionally disengaged, the potential impact of support was notably diminished, underscoring the importance of both physical proximity and emotional connection in facilitating sustained adherence. 

#### 3.4.3. Motivation: Emotional Encouragement from Family

Emotional encouragement emerged as a central driver of motivation. Participants reported that being supported, encouraged, and validated by family members strengthened their commitment to consistent MMS intake. This emotional support not only enhanced women’s belief in the personal and fetal health benefits of MMS but also sustained their motivation in the face of physical discomfort or forgetfulness:

“No matter how unwell I felt or how difficult it was, I had family helping motivate and advise me. So, I also felt more motivated.”

PW in Ratanakiri

Spousal involvement was often described as both practical and emotionally meaningful. However, a few participants noted that reminders could feel burdensome or nagging, particularly when delivered without sensitivity or after the supplement had already been taken, suggesting the importance of intentionality and empathy in supportive interactions.

“It is difficult to say. I felt that he only told me what he should tell me, but he didn’t really care about me.”

PW in Ratanakiri

“I felt nagging when I already took supplement and he would still call to remind me. On some days that I forgot, and he reminded me, I felt happy instead.”

PW in Kampot

Together, these findings illustrate that family support was perceived to function as a catalyst across all three COM-B domains—shaping habits, creating opportunity structures, and reinforcing motivation—which participants described as supporting their MMS adherence during pregnancy.

### 3.5. Tracking Calendar Intervention

The tracking calendar intervention was generally well received by participants, who consistently described it as visually appealing, appropriately sized, and easy to use in a home setting. This tool proved especially valuable for first-time mothers, who perceived it as a practical and trustworthy source of guidance on both prenatal supplement intake and healthy pregnancy practices. 

#### 3.5.1. Capability: Visual Tracking and Memory Support

Analyzed through the COM-B model, the calendar supported participants’ psychological capability by simplifying the process of daily MMS tracking.

“When taking the calendar home, it reminded me to take supplements every day.”

PW in Kampong Chhnang

“Easy to remember, so we wouldn’t be confused that today we already took supplements and tomorrow, we would take supplements again…we knew how many pills of supplements we had taken”

PW in Ratanakiri

The use of engaging images aided understanding among women with lower literacy, although some participants found certain illustrations unclear and in need of further explanation. Several participants recommended adding days of the week to the calendar layout to enhance clarity and better align the tool with their daily routine. These findings suggest that while the calendar format was broadly user-friendly, its effectiveness could be improved through refinements to visual design and labeling. 

#### 3.5.2. Opportunity: Environmental Cues in the Home

In terms of opportunity the calendar functioned as a visible environmental cue within the household.

“Sometimes, I forgot and when I turned to it [the calendar], I remembered that I forgot to take a supplement or I forgot to check off.”

PW in Takeo

Its placement in shared spaces sometimes encouraged family involvement in tracking, creating a supportive home environment where others could participate in or reinforce supplement-taking behavior.

“My husband read it and told me about what they didn’t allow me to do.”

PW in Kampot

However, some women found the action of marking their intake to be unnecessary, particularly if they were already in the habit of taking MMS regularly. Others admitted forgetting to record even when they had consumed the supplement, highlighting a perceived burden associated with the additional tracking task.

“For me, I didn’t forget to take supplements. I took supplements every day, but I forgot to write on the calendar…It was delayed on some days. I already took supplements for two days, but I forgot to write on [the] calendar. And I went back to write it down when I remembered.”

PW in Kampong Chhnang

#### 3.5.3. Motivation: Progress Monitoring and Goal Reinforcement

The calendar also provided motivational support and positive reinforcement for adherence to supplements. For many participants, checking off each day served as a reinforcing behavior, generating satisfaction, and a sense of accomplishment. The structure of the calendar—divided into clearly segmented spaces for marking intake—allowed women to visibly track their own progress, which was cited as a motivating factor in continuing daily supplement use.

“By counting, it felt like we passed to higher level”

PW in Kampot

The messaging on the calendar, emphasizing that MMS helps ensure a healthy, strong, and smart baby, further motivated women to adhere consistently. Participants recommended the tracking calendar would be especially valuable for women pregnant for the first time, as it provided key information on pregnancy dos and don’ts.

“Those who have their first pregnancy, they don’t know much. When they have this calendar, they will understand more.”

PW in Kampong Chhnang

Despite being ranked third in overall preference, participants’ accounts suggested that the calendar intervention still played a meaningful role in supporting MMS adherence by influencing all three components of behavior change outlined in the COM-B framework. It improved users’ ability to remember and monitor supplement use (capability), provided a constant physical signal that prompted action (opportunity), and encouraged goal completion by making progress visible and rewarding (motivation). Future iterations should focus on simplifying the tracking mechanism and enhancing visual clarity to ensure that the intervention remains accessible and effective across literacy levels.

### 3.6. Video Intervention

The video intervention consisted of short films depicting pregnant women and healthcare providers discussing MMS and healthy pregnancy practices. Participants reported trusting the content, as it was delivered by healthcare providers, and appreciated the conversational style, which felt natural and relatable.

#### 3.6.1. Capability: Improving Understanding Through Educational Videos

When assessed through the lens of the COM-B model, the video intervention influenced key drivers of behavior. From a capability standpoint, the videos enhanced participants’ understanding of MMS and pregnancy side effect management by providing clearly articulated and easily digestible information. This was especially beneficial for individuals with lower literacy levels, who could learn through a visual and auditory methods rather than written materials. Participants consistently reported high trust in the content, noting that information presented by healthcare providers felt credible and authoritative.

“I trusted it… because health professional clearly explained. So, I knew that the information that I received was good.”

PW in Kampong Chhnang

Participants indicated that the content addressed common pregnancy-related concerns and questions that mirrored their own experiences.

“What they said was similar to what we experienced making it easy to understand.”

PW in Kampot

“The information was clear and we could understand because the information in some video was about what we personally experienced and the information in some video was about what we never experienced because we haven’t reached the period of pregnancy.”

PW in Takeo

This alignment validated their feelings and provided reassurance, particularly around symptoms like morning sickness.

“The video made me understand that dizziness was the normal symptom of morning sickness”

PW in Kampot

Several women reported that hearing the messaging around MMS benefits and coping with pregnancy discomforts motivated them to adhere to daily supplementation and sustain healthy practices, even when feeling ill.

#### 3.6.2. Opportunity: Normalizing MMS Use via Relatable Stories

In terms of opportunity, the videos helped normalize MMS use by placing supplementation in familiar social contexts, creating a sense of peer support and shared experience. The conversational style of the videos, featuring interactions between pregnant women and healthcare providers, made the messages relatable, accessible, and engaging, increasing both attention and receptiveness.

“For me, before listening to information told by health professional, I felt nervous! Because it had many pills. Before, I only took 90 pills, but there were 180 pills of these supplements. When it was [the] delivery date, I wondered whether I should continue to take it or not. After watching the videos, health professional said although it was close to delivery date or after giving birth, I had to continue to take all of these supplements because it helped the baby a lot. So, I didn’t feel nervous anymore! I listened to what health professional advised”

PW in Kampong Chhnang

The presence of both healthcare providers and pregnant women in the videos also contributed to a socially supportive narrative, validating viewers’ own choices and reinforcing the importance of sustained adherence. Some participants expressed a desire for the videos to include more personal testimonials, specifically comparing their experiences of women using MMS versus traditional IFA.

#### 3.6.3. Motivation: Strengthening Confidence and Intention to Adhere

The videos offered emotional and cognitive prompts for participants. Highlighting the connection between MMS and healthy pregnancy outcomes instilled a sense of responsibility and encouragement, while new knowledge about recognizing and coping with common symptoms gave participants more confidence in navigating early pregnancy challenges.

“It was useful in helping to motivate us to take supplement regularly and to take supplement until there was no more left.”

PW in Takeo

“For me… ‘for mother and smart children’, I was interested in that word, and I had to take it every day. No matter how much I vomited, I had to take it.”

PW in Kampong Chhnang

However, a key limitation reported by participants was the timing of the intervention. Since the videos were shown after the calendar intervention, many women felt that they did not contain any new or additional information. This diminished their perceived utility, particularly among participants who were already familiar with the key messages. As a result, many participants perceived the videos as reinforcement rather than as a source of new learning or behavior change.

In conclusion, while the videos were not ranked as the most preferred or perceived as the most impactful intervention overall, participants’ narratives indicate that they played an important role in reinforcing key messages about MMS adherence and healthy pregnancy. Their credibility, relatability, and repeated emphasis on beneficial outcomes supported behavior change through enhanced knowledge and engagement.

## 4. Discussion

This mixed-methods study offers a detailed exploration of Cambodian women’s preferences and experiences with three distinct interventions designed to improve adherence to MMS during pregnancy. Among the interventions explored, family support emerged as the most preferred, followed by videos and the tracking calendar. Participants achieved a median adherence of 97% based on pill counts. Narratives from FGDs with women helped provide insight into the strategies that supported high adherence.

Family support emerged as an effective intervention for promoting MMS adherence, with participants consistently valuing the presence, encouragement, and practical assistance offered by husbands and other close family members. This finding is consistent with studies from India and Bangladesh, where family, particularly spousal involvement, significantly improved adherence to IFA supplementation through behaviors such as procuring tablets and providing daily reminders [21]. Further research from Peru, Zimbabwe, and Kenya underscores the role of spousal support in promoting adherence to micronutrient supplementation, highlighting the importance of engaging family members in behavior change initiatives [22,23,24].

A pregnant individual’s decisions and behaviors are greatly influenced by their family and cultures [25]. Contextually, family and cultural norms in Cambodia, similar to those in other Southeast Asian societies, position husbands as key decision-makers in household health matters [26]. A study in Bangladesh found that involving husbands in a maternal nutrition program significantly increased their wives’ intake of micronutrient supplements and dietary diversity during pregnancy [27,28]. The husbands’ increased awareness, knowledge, self-efficacy, and support contributed to these positive changes [27,28]. Therefore, it is critical that husbands are involved in ANC programming.

Operationalizing family support within ANC in Cambodia could draw on successful approaches from related contexts, such as community outreach sessions in India, decentralized ANC in Ethiopia, and home visits in Bangladesh. Suggestions from participants and the literature include structured incentives for spouse involvement in ANC [22], adherence support partners from social networks, and dedicated forums where family members can learn, share, and commit to supporting maternal nutrition [24]. Strengthening community platforms in partnership with ANC health systems aligns with other behavior change frameworks and successful IFA supplementation programs in other settings [29]. In Cambodia, partnerships with village health support worker groups could further reinforce family and community engagement in maternal supplement adherence efforts, beyond direct support from midwives. At the same time, family-based sessions are more resource-intensive than videos or calendars, requiring health worker time, coordination with family members, and sometimes additional costs for participants, so scalable models will likely need to embed family engagement within existing ANC and community platforms (for example, village health support groups and group ANC) and complement it with lower-intensity tools such as digital videos and simple tracking aids.

Videos and tracking calendars were ranked as less effective but still valuable interventions. These tools primarily enhanced pregnant individuals’ psychological capability and motivation by providing accessible education and structured reminders to sustain adherence. During the FGDs some participants highlighted how the information presented in the interventions, specifically the calendar and educational videos, would be especially important for women pregnant for the first time. Most of the participants in our study had been pregnant before, which could have influenced their perception of the importance of these interventions and their adherence to taking MMS. In urban settings, where close family may be less present day-to-day, strategies such as calendars and videos may have greater relative importance, whereas family-focused approaches remain relevant where nuclear families live together, which is still common in Cambodia.

Education-based strategies have shown to result in improved adherence and therefore would be helpful for first-time mothers. Specifically, short, education-focused videos represent a low-cost, easy-to-scale intervention that has been successful in other contexts [30]. Integrating these approaches into digital platforms can further enhance reach and sustainability. Distributing educational videos through social media and messaging applications offers a cost-effective way to deliver repeated, timely reminders and tailored information. While physical tracking calendars can support adherence, they entail recurring printing and distribution costs, which may limit long-term scalability.

In contrast, mobile applications can provide an integrated platform for both information and behavior tracking. A pregnancy-focused app endorsed by the Ministry of Health could deliver evidence-based content on antenatal care, nutrition, and MMS, while also allowing women to record daily supplement intake, receive automated reminders, and flag missed doses for follow-up by health workers. This approach has been shown to be effective in Tanzania in improving adherence to ANC WHO recommendations for a positive pregnancy [31]. While Nepal’s Mero Poshan app offers a useful model, as it was developed by the Ministry of Health to provide standardized maternal, neonatal, and nutrition messages and to strengthen communication between pregnant women, caregivers, and the health system [32,33].

### 4.1. Methodological Strengths and Rigor

This study’s strengths include its robust, multi-site design spanning four diverse Cambodian provinces and its use of a theory-driven analytical approach grounded in the COM-B model, ensuring comprehensive attention to capability, opportunity, and motivation. Data collection was conducted by trained local staff fluent in the language and context; all focus group discussions were audio-recorded and transcribed verbatim, with careful translation and verification to maintain fidelity. The research team engaged in iterative, collaborative coding and analysis procedures to enhance trustworthiness and transparency, regularly consulting with field researchers to ensure that interpretations accurately reflected the lived experiences of participants. 

### 4.2. Limitations

First, the sequential design, in which all participants received family support, the tracking calendar, and educational videos in a fixed order, may have introduced order effects and contamination, potentially influencing how later interventions were experienced and used. Therefore, the ranking should be interpreted cautiously, highlighting women’s relative preferences and perceived utility under these conditions, but they do not provide a definitive hierarchy of which intervention would be most effective if introduced alone or in a different sequence. Future studies could test each intervention independently, randomize the order of exposure, or evaluate an integrated adherence-support package to better isolate effects. Second, this study focused on individual and community-level factors and did not directly assess health system or structural determinants of MMS adherence, such as supply chain reliability, health worker workload and training, or institutional coordination, which are recognized barriers to IFA and MMS adherence in Cambodia and similar settings [9]. Third, given the close involvement of ANC staff, repeated contact with the research team, and the use of FGDs, participants’ responses are subject to potential social desirability bias, and reported preferences, perceived usefulness, and adherence behaviors may overestimate what would be observed in routine program conditions. Fourth, findings are most applicable to multiparous women in rural/urban settings and generalization to first-time pregnant women and urban women should be cautious and benefit from further piloting. And, lastly, because individual qualitative contributions were not linked to pill count data, it was not possible to stratify findings by adherence profile in this study, which limits our ability to compare intervention experiences across adherence strata and highlights an important area for future mixed-methods research.

## 5. Conclusions

This mixed-methods study shows that behavioral interventions—grounded in the COM-B framework—helped pregnant women in Cambodia adhere to daily MMS. Across four provinces, women reported that with clear information and a supportive home environment, taking MMS became a regular and manageable part of daily life.

Family support was the most valued and influential intervention, with husbands and other relatives playing a central role in reminding women, managing side effects, and encouraging continued use of MMS. These results align with findings from other settings: family members, particularly household decision-makers, have a significant impact on maternal nutrition. This underscores the importance of actively involving families in routine antenatal care and MMS programs in Cambodia.

Educational videos and tracking calendars were less frequently ranked first, but they still offered important added value by reinforcing key messages, improving understanding, and providing simple tools to help remember daily intake. Videos appeared particularly useful for women with lower literacy, whereas calendars were especially helpful for first-time mothers who were still establishing new routines during pregnancy. Together, the three interventions suggest that no single strategy is sufficient and that combining interpersonal, print, and digital approaches is likely to better address the different needs and preferences of pregnant women.

Given the high acceptability of MMS and the reported strong influence of family support, future MMS programs in Cambodia should prioritize strategies that actively engage husbands and family members, in addition to women themselves. An integrated, multilevel approach that combines family-focused, educational, and system-level strategies is recommended for further piloting and scale-up. Within such an integrated, multilevel approach, family engagement and strong health worker endorsement of MMS are likely to be essential elements, with lower-intensity tools such as calendars and educational videos serving as complementary supports that reinforce, rather than replace, these interpersonal components. To build on these exploratory findings, future quantitative or hybrid effectiveness–implementation studies are needed to assess the impact and scalability of these intervention packages on MMS adherence and maternal–infant outcomes. Engaging ANC stakeholders, leveraging locally embedded health workers, and maintaining rigorous monitoring and evaluation will be vital to ensuring that interventions are both contextually appropriate and sustainably effective for pregnant women across Cambodia.

## Figures and Tables

**Table 1:** Participant characteristics by province ^1^.

Characteristic	Kampong Chhnang (*n* = 9)	Takeo (*n* = 9)	Kampot (*n* = 9)	Ratanakiri (*n* = 9)
Age, year, mean ± SD	32 ± 5	26 ± 4	30 ± 5	28 ± 5
Marital status, married, *n* (%)	9 (100)	9 (100)	9 (100)	9 (100)
Gravidity, *n* (%)				
Primigravida	0 (0)	1 (11)	1 (11)	0 (0)
Multigravida	9 (100)	8 (89)	8 (89)	9 (100)
Multigravida, number of pregnancies, mean ± SD	3 ± 1	2 ± 1	3 ± 1	3 ± 1
Gestational age at first ANC visit, weeks, mean ± SD	8 ± 4	8 ± 2	10 ± 2	10 ± 3
Education completed, *n* (%)				
None	0 (0)	0 (0)	0 (0)	1 (11)
Primary	6 (67)	3 (33)	5 (56)	4 (44)
Secondary	0 (0)	2 (22)	4 (44)	1 (11)
Higher secondary	3 (33)	4 (44)	0 (0)	3 (33)

^1^ Total *n* = 36.

**Table 2:** Intervention ranking ^1^.

	Kampong Chhnang (*n* = 7)	Takeo (*n* = 8)	Kampot (*n* = 9)	Ratanakiri (*n* = 7)	All (*n* = 31)
Family support	1 (100%)	1 (50%)	1 (55%)	2 (43%)	1 (61%)
Calendar	3 (71%)	3 (75%)	2 (33%)	3 (86%)	3 (61%)
Video	2 (71%)	2 (50%)	3 (67%)	1 (57%)	2 (48%)

^1^ Overall rank (% of participants that ranked the same way). Participant instructions: Rank the interventions on a scale of 1 to 3, with 1 being most effective and 3 being least effective at helping you take MMS daily and follow healthy pregnancy practices.

**Table 3:** Illustrative COM-B-informed coding tree for family support and MMS adherence ^1^.

COM-B Domain	Thematic Category	Description
MOTIVATION (automatic & reflective)	Overall impression of family involvement	Women’s emotional responses ranged from positive (feeling supported and encouraged) to negative (feeling burdened or stressed). Some reported neutral responses, while a few gave no substantive feedback.
	Communication frequency & style	Excessive reminders felt like nagging, while “just enough” reminders were helpful. Communication styles ranged from supportive and gentle to nagging, angry, or upset; some family members were accepting and calm.
	Motivation, encouragement & self-regulation	Family support enhanced emotional well-being and encouraged consistent use of MMS. Some women demonstrated self-reliant routines independent of reminders and valued emotional support and patience over constant reminders.
CAPABILITY (physical & psychological)	Family involvement in MMS support	Family members provided varying levels of support: active participation (reminders, supplement preparation), limited involvement due to distance or work constraints, and a mix of involvement across family members.
	Routine development & reminder strategies	Family reminders helped establish and maintain daily supplement routines. Some women showed improved consistency, while others reported no change or worse adherence due to over-reliance on external reminders.
	Practical help, advice & knowledge	Families received health advice on nutrition, physical activity, and reducing workload. Household support (cooking, laundry, shopping) reduced women’s burden. Family members who attended education sessions provided more informed support.
OPPORTUNITY (social)	Comfort with different family members	Comfort levels varied among family members and according to their involvement style. Husbands and mothers generally created comfort when engaged supportively; fathers were appreciated for teaching roles. Some women felt comfortable with specific relatives but not others.
	Social barriers and tensions	Family involvement sometimes created conflict, particularly with controlling or nagging styles, which reduced adherence. However, many women experienced no tension, indicating that respectful support strengthened relationships.
	Future family-focused support design	Women recommended: enhancing family knowledge about MMS benefits; training on gentle communication; improving session accessibility and timing; focusing on relevant topics; and designing interventions that fit family schedules.

^1^ This narrative table presents an illustrative subset of the complete coding tree for family support FGDs, organized by COM-B domains (Motivation, Capability, Opportunity) and key thematic categories. Each row synthesizes multiple individual codes into summaries of patterns and insights. The full detailed codebook, with individual codes, example quotes, and coding strategy, is provided in the Supplementary Materials.

## Data Availability

Data described in the manuscript will be made available upon reasonable request pending application and approval by the principal investigator.

## Supplementary Materials

The following supporting information can be downloaded at: https://www.mdpi.com/article/10.3390/nu18040583/s1. Files: Codebook Family Support; Codebook for Calendar Tracking; Codebook for MMS videos; Family Support Information Session Handout; FGD Guide Intervention Family Support; FGD Guide Intervention Videos; FGD Guide Intervention Wall Calendar; Tracking Calendar.

## Abbreviations

The following abbreviations are used in this manuscript:

ANC	Antenatal care
COM-B	Capability, Opportunity, Motivation–Behavior
FGD	Focus group discussion
IFA	Iron folic acid
MMS	Multiple micronutrient supplements
UNIMMAP	United Nations International Multiple Micronutrient Antenatal Preparation
WHO	World Health Organization
