# Supplementary material for: Exploring Behavioral Interventions to Enhance Adherence to Multiple Micronutrient Supplementation Among Pregnant Women in Cambodia: A Mixed-Methods Study
Source: Nutrients. 2026 Feb 10;18(4):583. doi: 10.3390/nu18040583 (PMC12943067; doi:10.3390/nu18040583)
Supplement: Supplementary file 1 [file nutrients-18-00583-s001.zip › Supplementary Materials/Codebook Family Support.pdf]

Summary COM-B–Informed Codebook for Family Support During Pregnancy and MMS Adherence

COM-B domain (main – sub domain)	Thematic category	Code	Description	Example quote	Strategy
Motivation – automatic	Overall impression of family involvement	1-OI-POSITIVE	Pregnant woman expresses overall positive feelings about family involvement in her pregnancy and MMS use (e.g., feeling supported, cared for, or motivated by family).	"I felt supported when my family reminded me about my supplements."	Deductive
Motivation – automatic	Overall impression of family involvement	1-OI-NEGATIVE	Pregnant woman expresses overall negative feelings about family involvement (e.g., feeling pressured, annoyed, burdened, or stressed by family reminders or behaviours).	"The constant reminders from my family felt burdensome."	Deductive
Motivation – reflective	Overall impression of family involvement	1-OI-NEUTRAL	Family involvement is described as acceptable but not especially influential (e.g., neutral, mixed, or ambivalent views about whether it helps MMS adherence).	"I didn't mind the reminders, but they didn't make much difference."	Deductive
Motivation – automatic	Overall impression of family involvement	1-OI-NO RESPONSE	Woman gives no substantive response or indicates she has no particular feelings about family involvement.	"No."	Inductive

COM-B domain (main – sub domain)	Thematic category	Code	Description	Example quote	Strategy
Motivation – automatic	Communication frequency	4-CF-EXCESSIVE	Reminder frequency is perceived as too frequent or intrusive (e.g., “nagging”), leading to irritation or reduced motivation.	“They reminded me so often that it became annoying.”	Deductive
Motivation – reflective	Communication frequency	4-CF-HLP-KROB KRORN	Reminder frequency is perceived as appropriate and helpful (e.g., “just enough”) to support remembering daily supplements without becoming bothersome.	“The daily reminders were just right and really helped me remember.”	Deductive
Motivation – automatic	Communication frequency	4-CF-MIXED	Reminder frequency is perceived as both sufficient and sometimes too much (helpful but also repetitive or nagging), resulting in mixed emotional reactions.	“Sometimes, I felt nagging and sometimes, I felt happy that he worried about me.”	Inductive
Capability – psychological	Communication frequency	4-CF-INS	Reminder frequency is perceived as too low to reliably support adherence (e.g., woman reports still forgetting despite some reminders).	“I wish they had reminded me more often; I still forgot sometimes.”	Deductive
Motivation – reflective	Communication style	5-CS-HELPFUL	Family members use a supportive, gentle, or caring tone and language that the woman perceives as helpful	“My family's gentle reminders were very helpful.”	Deductive

COM-B domain (main – sub domain)	Thematic category	Code	Description	Example quote	Strategy
			for her supplement adherence and well-being.		
Motivation – reflective	Communication style	5-CS-NAGGING/THUNH	Family communication is perceived as nagging, cold, or obligation-driven (e.g., scolding, repetitive questioning), which can undermine motivation.	"The constant reminders felt like nagging after a while."	Deductive
Motivation – reflective	Communication style	5-CS-UPSET	Family communication about MMS or pregnancy becomes angry, irritated, or blaming, leading to tension or discomfort.	"Sometimes he gets mad at me."	Inductive
Motivation – reflective	Communication style	5-CS-ACCEPTING/TOTOUL YORK	Family responds calmly and non-reactively even when the woman is irritable or non-adherent (accepting, patient responses that avoid conflict).	"Sometimes, I rudely talked to him, but he didn't mind or get angry with me."	Inductive
Motivation – reflective	Self-reliant routine	3-SR-SELF	Woman positions herself as primarily responsible for remembering and managing supplement intake, with little reliance on external reminders.	"I still remembered taking supplement even he forgot to remind me… I never forgot."	Inductive

COM-B domain (main – sub domain)	Thematic category	Code	Description	Example quote	Strategy
Motivation – automatic<br><br>Capability – physical/psychological	Emotional well-being impact	7-PI-EMOTIONAL	Family involvement positively affects mood and emotional state (e.g., feeling loved, reassured, or less lonely), which in turn supports ongoing MMS use.	"I felt happy when my family came to the session. When they came back, they shared me many things."	Deductive / Inductive
Motivation – reflective	Encouragement	7-PI-ENCOURAGED	Family support is described as encouraging or motivating (e.g., expressions of care, concern, or praise) that strengthens intention to adhere to MMS.	"It encouraged me because they helped to remind me and it means that they cared about my health."	Inductive
Motivation – reflective	Recommendations – communication	12-RFM-COMMUNICATE	Woman suggests preferred ways for family to communicate support (e.g., asking about her feelings first, using kind or respectful language).	"They should ask how we're feeling before giving reminders."	Deductive
Motivation – reflective	Recommendations – emotional	12-RFM-EMOTIONAL	Woman recommends that family provide understanding, patience, and empathy rather than criticism or pressure when supporting MMS use.	"Showing understanding and patience is more important than constant reminders."	Deductive

COM-B domain (main – sub domain)	Thematic category	Code	Description	Example quote	Strategy
Motivation – automatic	Recommendations – reminders	12-RFM-SUPPLEMENT REMINDER	Woman explicitly recommends that family continue or start reminding her about taking supplements as a valued form of support.	"Oun, did you take supplements yet?"	Inductive
Capability – physical & psychological	Family involvement in support	2-FI-ACTIVE	One or more family members are actively involved in practical and/or emotional support (e.g., preparing supplements, monitoring intake, encouraging rest and diet).	"My husband actively reminded me every day and even prepared my supplements."	Deductive & Inductive
Capability – physical & psychological	Family involvement in support	2-FI-HUSBAND	Husband is specifically described as actively involved (e.g., reminding daily, reducing her workload, bringing or preparing supplements/food).	"Ever since my husband knew that I was pregnant, he didn't let me do much work. He always reminded me to take supplements."	Inductive
Capability – psychological	Family involvement in support	2-FI-MOTHER	Mother is specifically described as actively involved (e.g., checking daily intake, prompting her to take supplements or eat).	"My mother asked me every day. She asked whether I already took supplements."	Inductive

COM-B domain (main – sub domain)	Thematic category	Code	Description	Example quote	Strategy
Capability – psychological	Family involvement in support	2-FI-FATHER	Father is specifically described as actively involved (e.g., reminding, advising, or teaching her about health behaviours).	"He also reminded me every day."	Inductive
Capability – psychological	Family involvement in support	2-FI-LIMITED	Family involvement exists but is constrained (e.g., infrequent reminders, distance, work commitments), limiting their practical or emotional support.	"My family members rarely mentioned the supplements unless I brought it up."	Deductive
Capability – physical	Family involvement in support	2-FI-MIXED	Different family members show varying levels of involvement (e.g., one person highly engaged while others rarely help), leading to uneven support.	"My mother was very involved, but my siblings hardly participated at all."	Deductive
Capability – physical	Routine development	7-PI-ROUTINE	Family support (e.g., consistent reminders, shared routines) helps the woman establish or maintain a regular MMS-taking habit.	"Yes. He helped to remind me when I forgot. So, I regularly take supplements every day. I never forgot."	Deductive

COM-B domain (main – sub domain)	Thematic category	Code	Description	Example quote	Strategy
Capability – physical & psychological	Nutrition and health advice	7-PI-ADVICE	Family provides broader health or lifestyle advice (e.g., about eating, rest, or avoiding risky behaviours) perceived as beneficial in pregnancy.	"He tried to remind me to eat nutritious food as well as things to be avoided."	Inductive
Capability – physical & psychological	Nutrition and health advice	7-PI-NUTRITIOUS EATING	Family specifically encourages increased intake of nutritious foods (e.g., vegetables, fruits, or other nutrient-dense items).	"He also told me to eat vegetables because it had many benefits."	Inductive
Capability – physical & psychological	Workload advice	7-PI-REDUCE HEAVY WORK	Family encourages or enforces reduction of heavy physical labour or household tasks to protect maternal health.	"He didn't allow me to lift heavy stuff."	Inductive
Capability – psychological	Practical reminders	7-PI-REMINDERS	Family provides direct reminders about when and how to take MMS (e.g., daily verbal prompts, timing linked to meals or bedtime).	"When my mother came to visit me… she told me to take it before I went to sleep."	Inductive
Capability – physical & psychological	Practical support with chores	7-PI-FAMILY CHORES	Family members reduce the woman's domestic workload (e.g., cooking, cleaning, shopping, laundry) to support her health and adherence.	"When I was feeling unwell… he helped to do laundry."	Inductive

COM-B domain (main – sub domain)	Thematic category	Code	Description	Example quote	Strategy
Capability – physical & psychological	Physical well-being	7-PI-PHYSICAL	Woman attributes improvements in physical health (e.g., more energy, fewer symptoms) to support received from family.	“With my family's support, I felt more energetic and healthier.”	Deductive
Capability – physical & psychological	Family knowledge	7-PI-FAMILY KNOWLEDGE	Family members are described as having gained understanding about pregnancy, MMS, or maternal needs, which shapes how they provide support.	“When he got to join the session… he knew that pregnant woman faced a lot of difficulties.”	Inductive
Capability – physical	Change in supplement routine	9-PI-ROUTINE	Woman reports that her supplement-taking routine improved (e.g., more regular, fewer missed doses) following family involvement or the intervention.	“I’ve been much more consistent with taking my supplements since my family got involved.”	Deductive
Capability – physical	Change in supplement routine	9-CR-UNCHANGED	Woman reports that her supplement-taking routine remained essentially the same despite family involvement.	“Their reminders didn't really change my supplement-taking habits.”	Deductive
Capability – physical	Change in supplement routine	9-CR-WORSE	Woman reports that her routine worsened (e.g., more forgetfulness) due to over-reliance on family	“I actually started forgetting more often because I	Deductive

COM-B domain (main – sub domain)	Thematic category	Code	Description	Example quote	Strategy
			reminders or other aspects of involvement.	relied too much on their reminders.”	
Capability – physical	Recommendations – practical support	12-RFM-PRACTICAL	Woman recommends practical help from family (e.g., food preparation, household tasks, transport to clinic) as more useful than reminders alone.	“Help with household chores can be more supportive than just verbal reminders.”	Deductive
Capability – psychological	Future design – knowledge	11-FID-KNOWLEDGE	Suggestions that future interventions should strengthen family knowledge about MMS and pregnancy (e.g., more education, clearer explanations).	“It would be helpful if they understood more about why the supplements are important.”	Deductive
Capability – psychological	Future design – approach	11-FID-APPROACH	Suggestions to improve how family support is delivered (e.g., training on gentle communication, involving key relatives, framing messages positively).	“Teaching family members how to provide gentle reminders would be beneficial.”	Deductive
Capability – physical	Future design – resources	11-FID-RESOURCES	Suggestions for additional tools or resources (e.g., reminder tools, apps, materials) to help families support MMS adherence.	“Providing families with a tracking app could make the	Deductive

COM-B domain (main – sub domain)	Thematic category	Code	Description	Example quote	Strategy
				reminders more effective.”	
Opportunity – social	Comfort with husband	6-CL-HUS-POS	Woman feels comfortable and positive about husband’s involvement in reminders and support.	“I felt most comfortable when my husband reminded me.”	Deductive
Opportunity – social	Comfort with husband	6-CL-HUS-NEUT	Woman is neutral or ambivalent about husband’s involvement, perceiving it as neither strongly helpful nor problematic.	“I didn't mind my husband’s reminders, but he didn't make much difference.”	Deductive
Opportunity – social	Comfort with mother-in-law	6-CL-MIL-POS	Woman feels comfortable and supported by mother-in-law’s involvement in her care and MMS use.	“I felt most comfortable when my mother-in-law reminded me.”	Deductive
Opportunity – social	Comfort with mother-in-law	6-CL-MIL-NEG	Woman feels uncomfortable, criticised, or constrained by mother-in-law’s involvement.	“I did not feel comfortable when my mother-in-law reminded me.”	Deductive
Opportunity – social	Comfort with mother-in-law	6-CL-MIL-NEUT	Woman reports neutral or mixed comfort with mother-in-law’s involvement (neither clearly positive nor clearly negative).	“I didn't mind my mother-in-law's reminders, but	Deductive

COM-B domain (main – sub domain)	Thematic category	Code	Description	Example quote	Strategy
				she didn't make much difference.”	
Opportunity – social	Comfort with mother	6-CL-MOTH-POS	Woman feels comfortable and positively supported by her mother’s involvement.	“The way she speaks was sweet and gentle.”	Inductive
Opportunity – social	Comfort with mother	6-CL-MOTH-NEG	Woman feels uncomfortable or unhappy about the way her mother provides reminders or support.	“I did not feel comfortable when my mother reminded me.”	Deductive
Opportunity – social	Comfort with mother	6-CL-MOTH-NEUT	Woman has neutral or ambivalent feelings about her mother’s involvement.	“I didn't mind my mother’s reminders, but she didn't make much difference.”	Deductive
Opportunity – social	Comfort with father	6-CL-FATH-POS	Woman feels comfortable and supported by her father’s involvement (e.g., teaching, explaining, or guiding).	“My father cared about me more… he explained me everything.”	Inductive
Opportunity – social	Comfort with father	6-CL-FATH-NEG	Woman feels uncomfortable or dissatisfied with her father’s involvement.	“I did not feel comfortable when my father reminded me.”	Deductive

COM-B domain (main – sub domain)	Thematic category	Code	Description	Example quote	Strategy
Opportunity – social	Comfort with father	6-CL-FATH-NEUT	Woman is neutral or ambivalent about her father's involvement.	"I didn't mind my father's reminders, but he didn't make much difference."	Deductive
Opportunity – social	Comfort with other relatives	6-CL-OTH-POS	Woman feels comfortable and positively supported by other relatives (e.g., siblings, in-laws, aunts).	"My sister's support was the most comfortable for me."	Deductive
Opportunity – social	Comfort with other relatives	6-CL-OTH-NEG	Woman feels uncomfortable or disagrees with how other relatives provide support or reminders.	"My sister in law's support was the most disagreeable for me."	Deductive
Opportunity – social	Comfort with other relatives	6-CL-OTH-NEUT	Woman reports neutral or limited feelings (neither strongly positive nor negative) about other relatives' involvement.	"I didn't mind my aunt's reminders, but it didn't make much of a difference for me."	Deductive
Opportunity – social & Motivation – reflective	Challenges experienced	8-CE-TENSION	Family involvement leads to arguments, strain, or interpersonal tension related to MMS or pregnancy behaviours.	"Sometimes the reminders led to arguments with my spouse."	Deductive

COM-B domain (main – sub domain)	Thematic category	Code	Description	Example quote	Strategy
Opportunity – social & Motivation – reflective	Challenges experienced	8-CE-UNHELPFUL	Family involvement is described as unhelpful or counterproductive (e.g., style or content of support reduces willingness to adhere).	"Their way of reminding me actually made me less inclined to take the supplements."	Deductive
Motivation – reflective	Self-reliant routine	8-CE-NO CHALLENGE	Woman explicitly reports no tension or challenges linked to family involvement.	"I didn't feel tense."	Inductive
Opportunity – social	Future design – accessibility	11-FID-ACCESSIBLE	Suggestions to make family-focused sessions easier to attend (e.g., better scheduling, advance notice, aligning with work).	"I want him to join, but he seems to have no time… you have to inform me first so that I can schedule with him."	Inductive
Opportunity – social	Future design – focus	11-FID-REMOVE	Suggestions to reduce or remove certain topics (e.g., diet) to minimise burden on family or focus on what is most useful.	"I can take care on my own eating because I want to give them more time to do their work or business."	Inductive

COM-B domain (main – sub domain)	Thematic category	Code	Description	Example quote	Strategy
Motivation – automatic<br><br>Capability – physical/psychological	Feeling cared for	7-PI-CARED FOR	Woman feels more cared for, valued, and protected by family after the intervention, often linked to concrete supportive actions.	“After he joined the session, he cared about me a lot.”	Inductive
Motivation – reflective<br><br>Capability – physical/psychological	Self-reliant routine	10-BD-BENEFIT	Woman judges that overall benefits of family involvement (e.g., support, reminders, reduced work) are greater than any drawbacks.	“Despite some annoyances, overall, their support was more helpful than not.”	Deductive
Motivation – reflective<br><br>Capability – physical/psychological	Benefits vs drawbacks	10-BD-DRAW	Woman judges that stress, conflict, or burden from family involvement outweigh any benefits.	“The stress from their involvement outweighed any benefits of their reminders.”	Deductive
Motivation – reflective<br><br>Capability – physical/psychological	Benefits vs drawbacks	10-BD-BALANCED	Woman describes a roughly equal mix of positive and negative aspects of family involvement, with no clear dominance.	“There were both good and bad aspects, but neither really outweighed the other.”	Deductive

COM-B domain (main – sub domain)	Thematic category	Code	Description	Example quote	Strategy
Motivation – reflective	Future design – no change	11-FID-NO CHANGES	Woman reports being satisfied with the current design and offers no suggestions for change.	“No, I think this was enough because they always reminded me.”	Inductive
