# Supplementary material for: Exploring Behavioral Interventions to Enhance Adherence to Multiple Micronutrient Supplementation Among Pregnant Women in Cambodia: A Mixed-Methods Study
Source: Nutrients. 2026 Feb 10;18(4):583. doi: 10.3390/nu18040583 (PMC12943067; doi:10.3390/nu18040583)
Supplement: Supplementary file 1 [file nutrients-18-00583-s001.zip › Supplementary Materials/Codebook for Calendar Tracking.pdf]

Summary COM-B–Informed Codebook for Calendar to Support MMS Adherence

COM-B domain (main – subdomain)	Thematic category	Code	Description	Example quote	Strategy
Capability – psychological<br><br>Opportunity – physical; Motivation – reflective	1. Calendar usage – tracking	1.1-SUPP_ TRACK	Any mention of using, partially using, or not using the calendar to track MMS intake (e.g., checking off days, looking at ticks, or explicitly not engaging with it).	"I checked off the tracker"	Deductive
Capability – psychological<br><br>Opportunity – physical;<br><br>Motivation – automatic	1. Calendar usage – frequency	1.2-FREQUENCY	How often the participant reports referring to or reading the calendar, whether as a visual reminder and/or to tick off doses. Use this as a parent code when frequency is discussed but not clearly high/moderate/low.	"I marked the calendar every day after taking my supplement"	Deductive
Capability – psychological Opportunity – physical	1. Calendar usage – frequency	1.2A-FREQ_HIGH	Participant reports using the tracker almost every day or every day (e.g., consistently checking and marking after taking MMS).	"I marked the calendar every day after taking my supplement"	Deductive

COM-B domain (main – subdomain)	Thematic category	Code	Description	Example quote	Strategy
Motivation – automatic					
Capability – psychological<br><br>Opportunity – physical;<br><br>Motivation – automatic	1. Calendar usage – frequency	1.2B-FREQ_ MODERATE	Participant reports using the tracker some days (e.g., 3–4 times per week), but frequently forgets to look at it or tick off doses.	"I marked the calendar sometimes, but would miss several days"	Deductive
Capability – psychological<br><br>Opportunity – physical;<br><br>Motivation – automatic	1. Calendar usage – frequency	1.2C-FREQ_ LOW	Participant reports rarely or never using the tracker (e.g., only 1–2 times per week or not at all).	"I didn't ever use the tracking tool."	Deductive
Capability – psychological;<br><br>Motivation – reflective	1. Calendar usage – perceived usefulness	1.3- USEFULNESS	Overall judgment of the calendar as a helpful or unhelpful pregnancy resource (e.g., supporting knowledge, routine, or confidence) including both positive and negative views.	"The calendar was very helpful throughout my pregnancy" or "I didn't find the calendar useful because..."	Deductive

COM-B domain (main – subdomain)	Thematic category	Code	Description	Example quote	Strategy
Capability – psychological;<br><br>Motivation – reflective	1. Calendar usage – placement	1.4-PLACEMENT	Any mention of where the calendar was placed or stored (e.g., bedroom, kitchen, bathroom) and how this location related to remembering to take MMS.	–	Inductive
Capability – psychological	1. Calendar usage – placement	1.4A-PLACE_ SLEEPING AREA	Calendar placed near the sleeping area (e.g., headboard, wall near bed), often to link seeing it with nightly or morning routines.	"I put it beside my bed"	Inductive
Capability – psychological	1. Calendar usage – placement	1.4B-PLACE_ EATING AREA	Calendar placed near the eating area (e.g., kitchen, dining table), often to link it with meals and pill-taking.	"I put it close to the table where we eat"	Inductive
Capability – psychological	1. Calendar usage – placement	1.4C-PLACE_ OTHER	Calendar placed in other locations (e.g., bathroom, living room) that do not clearly map to sleeping or eating spaces.	"I put it in my bathroom"	Inductive
Capability – psychological<br><br>Motivation – automatic	2. Design and visual elements – colours	2.1-COLOR_ SCHEME	Reactions to the colour choices of the calendar (e.g., bright, attractive, dull, confusing) and their influence on attention or appeal.	"I liked the bright colors" or "The colors were too dull"	Deductive
Capability – psychological	2. Design and visual	2.2-LAYOUT_ FORMAT	Comments on the layout and structure (e.g., weekly/monthly	"The weekly layout was easy	Deductive

COM-B domain (main – subdomain)	Thematic category	Code	Description	Example quote	Strategy
Opportunity – physical	elements – layout		format, grid, size), and how easy or difficult it was to navigate or hang/place.	to follow" or "I wish it was in a different format"	
Capability – psychological	2. Design and visual elements – images	2.3-IMAGES_ ICONS	Feedback on pictures and icons (e.g., clarity, cultural fit, recognisability) and whether they helped understanding or caused confusion.	"The pictures were helpful" or "I couldn't understand some of the illustrations"	Deductive
Capability – psychological	2. Design and visual elements – text	2.4-TEXT_ FEATURES	Comments on text size, font, language and readability (e.g., too small, easy to read, liked headings).	"The font was too small to read easily" or "I liked the bold headings"	Deductive
Capability – psychological	3. Information & content – clarity	3.1-CLARITY_ INFO	Perceived clarity of information (text and icons) in the calendar (e.g., easy to understand vs confusing wording, symbols, or instructions).	"The icons were easy to understa(nd" or "I was confused by some of the terms used"	Deductive
Capability – psychological; Opportunity – physical	3. Information & content – trimester focus	3.2-TRIMESTER_ SPECIFIC_CO NTENT	Feedback on information specific to different trimesters (e.g., which trimester content was most useful, where more/less detail was needed).	"The third trimester information was most useful to me" or "I wish	Deductive

COM-B domain (main – subdomain)	Thematic category	Code	Description	Example quote	Strategy
				there was more detail for the first trimester”	
Motivation – reflective	3. Information & content – relevance	3.3-CONTENT_RELEVANCE	Perceptions of how relevant and personally useful the information was (e.g., confirming what she already knew vs providing new, actionable guidance).	“The information helped me to know my symptoms were typical during pregnancy” or “The information wasn’t helpful as I already knew this from other sources”	Deductive
Motivation – reflective	3. Information & content – MMS benefit framing	3.3A-MOTIVATION_BABY_SMART	Mentions that MMS helps the baby to be “smart” or intelligent, used as a motivation for adherence by the woman or her family.	“Knowing the MMS helps the baby be smart encouraged me to keep taking it”	Inductive
Motivation – reflective	3. Information & content – MMS benefit framing	3.3B-MOTIVATION_STRONG_HEALTHY	Mentions that MMS helps the baby to be strong or healthy, motivating the woman or her family to support adherence.	“My husband wanted me to keep taking MMS for our baby to be healthy”	Inductive

COM-B domain (main – subdomain)	Thematic category	Code	Description	Example quote	Strategy
Motivation – reflective	3. Information & content – MMS benefit framing	3.3C-MOTIVATION_OTHER	Mentions of other perceived benefits of MMS (e.g., caring for own body, general health) that motivate adherence, beyond "smart" or "strong/healthy" baby.	"Taking MMS motivated me to take care of my body better"	Inductive
Capability – psychological<br><br>Motivation – reflective	3. Information & content – gaps	3.4-CONTENT_GAPS	Mentions of missing, insufficient, or desired additional information (e.g., breastfeeding, symptom management, more detail on nutrition or danger signs).	"I wish it had included information about breastfeeding" or "There should be more about managing pregnancy symptoms"	Deductive
Capability – psychological<br><br>Opportunity – physical;<br><br>Motivation – automatic	4. Effectiveness as reminder	4.1-POS_EFFECTIVENESS_REMINDER	Positive statements that the wall calendar helped them remember to take MMS (e.g., seeing it acted as a cue or prompt).	"Seeing the calendar on my wall reminded me to take my vitamins"	Deductive
Capability – psychological	4. Effectiveness as reminder	4.2-NEUTRAL_EFFECTIVENESS_REMINDER	Calendar is visible but perceived as having little or no impact on remembering to take MMS (e.g., still forgetting despite seeing it).	"Even though I saw the wall calendar each	Deductive

COM-B domain (main – subdomain)	Thematic category	Code	Description	Example quote	Strategy
Opportunity – physical<br><br>Motivation – reflective				night; I still would forget.”	
Capability – psychological<br><br>Motivation – reflective	4. Effectiveness as a reminder	4.3-ALTERNATIVE_ REMINDERS	Use of other cues or reminder systems instead of, or alongside, the calendar (e.g., phone alarms, family reminders).	“I relied more on my phone alarm” or “My husband reminded me instead of using the calendar”	Deductive
Capability – psychological<br><br>Opportunity – physical;<br><br>Motivation – automatic	5. Behavioral impact – MMS adherence	5.1-POS_EFFECTIVENESS_ REMINDER	(Same as 4.1) Positive references to the calendar functioning as an effective reminder that supported regular MMS intake.	“Seeing the calendar on my wall reminded me to take my vitamins”	Deductive
Capability – psychological<br>Opportunity – physical;<br>Motivation – reflective	5. Behavioral impact – MMS adherence	5.2-NEUTRAL_EFFECTIVENESS_REMINDER	(Same as 4.2) Calendar has little/no perceived impact on actual MMS-taking behaviour, even if seen regularly.	“Even though I saw the wall calendar each night; I still would forget.”	Deductive

COM-B domain (main – subdomain)	Thematic category	Code	Description	Example quote	Strategy
Capability – psychological;<br><br>Motivation – reflective	5. Behavioral impact – diet	5.3-DIETARY_CHANGES_ POS	Reported positive changes in diet or food choices that the woman attributes to the calendar (e.g., eating more nutrient-rich foods).	"I started eating more iron-rich foods after reading the calendar"	Deductive
Capability – psychological<br><br>Motivation – reflective	5. Behavioral impact – other behaviors	5.4-OTHER_BEHAVIORAL_ CHANGES	Reported changes in daily routines, self-care, rest, or physical activity due to calendar messages.	"I started taking more rest breaks as suggested in the calendar" or "I followed the exercise guidelines from the calendar"	Deductive
Capability – psychological<br><br>Motivation – reflective	5. Behavioral impact – family	5.5-FAM_BEHAVIORAL_ CHANGES	Calendar is described as influencing behaviour of other family members (e.g., their diet, support, or health practices).	"My husband started to eat healthier after I took the calendar home"	Inductive
Capability – psychological;<br><br>Motivation – reflective	6. Comparison to other resources – health providers	6.1-COMPARE_HEALTH_ PROVIDER	Comparisons between calendar information and advice from healthcare providers (e.g., consistency, trust, added value).	"The calendar had similar information to what my doctor told me" or "I trusted my doctor's advice	Deductive

COM-B domain (main – subdomain)	Thematic category	Code	Description	Example quote	Strategy
				more than the calendar”	
Capability – psychological;<br><br>Motivation – reflective	6. Comparison to other resources – family	6.2-COMPARE_FAMILY_ ADVICE	Comparisons between calendar information and advice from family members (e.g., alignment, contradictions, validation).	“My mother gave different advice than what was in the calendar” or “The calendar confirmed what my mother-in-law told me”	Deductive
Capability – psychological<br><br>Motivation – reflective	6. Comparison to other resources – other media	6.3-COMPARE_ OTHER	Comparisons between the calendar and other sources (e.g., apps, internet, books) in terms of detail, convenience, and usefulness.	“I found similar information online, but the calendar was more convenient” or “My pregnancy app was more detailed than the calendar”	Deductive
Opportunity – physical;<br><br>Motivation – reflective	7. Economic aspects – willingness to pay	7.1-WILLINGNESS _ TO_PAY	Any statements about willingness or unwillingness to pay for the calendar and reasons for that decision.	–	Deductive

COM-B domain (main – subdomain)	Thematic category	Code	Description	Example quote	Strategy
Opportunity – physical;<br><br>Motivation – reflective	7. Economic aspects – willingness to pay	7.1A-HIGH_WILL_PAY	Participant indicates a relatively high willingness to pay, viewing the calendar as worth the cost.	"I would buy it for 4,000 Riels because it's worth it"	Deductive
Opportunity – physical<br><br>Motivation – reflective	7. Economic aspects – willingness to pay	7.1B-LOW_WILL_PAY	Participant indicates low or no willingness to pay, viewing the calendar as too expensive or low priority.	"I wouldn't pay that amount because it's too expensive"	Deductive
Capability – psychological<br><br>Motivation – reflective	7. Economic aspects – perceived value	7.2-PERCEIVED_VALUE	Perceptions of the calendar's value relative to other things they might purchase (e.g., food, other items), including perceived longevity or usefulness.	"If I were to buy something to eat it would be gone but this has longer usefulness to me" or "I don't think it provides enough value to justify the cost"	Deductive
Capability – psychological;<br><br>Motivation – reflective	8. Suggestions for improvement – content	8.1-CONTENT_IMPROVEMENT	Suggestions to improve or expand the information or messages (e.g., more detail on symptoms, nutrition, danger signs).	"Include more information about managing common pregnancy problems" or "The nutrition advice	Deductive

COM-B domain (main – subdomain)	Thematic category	Code	Description	Example quote	Strategy
				should be more specific”	
Capability – psychological;<br><br>Motivation – reflective	8. Suggestions for improvement – design	8.2-DESIGN_IMPROVEMENT	Suggestions to improve visual or physical design (e.g., larger images, brighter colours, clearer icons).	“Make the images larger” or “Use brighter colors to make it more attractive”	Deductive
Capability – psychologica<br><br>Opportunity – physical	8. Suggestions for improvement – format	8.3-FORMAT_CHANGES	Suggestions for alternative formats (e.g., larger wall poster, booklet, different size) or ways to present the same content.	“I would prefer if it was a larger poster on the wall” or “I want it to be in a book format”	Deductive
Opportunity – social;<br><br>Motivation – reflective	9. Community impact – perceived usefulness	9.1-COMMUNITY_USEFULNESS	Perceptions about whether other women in the community would find the calendar useful or be likely to use it.	“Other women in my village would benefit from this calendar” or “I don't think many women here would use it”	Deductive
Opportunity – social<br><br>Motivation – reflective	9. Community impact – recommendation	9.2-RECOMMENDATION_INTENTIONS	Whether the participant would recommend the calendar to others and why (e.g., perceived benefits or limitations).	“I would recommend this to my pregnant sister” or “I wouldn't tell	Deductive

COM-B domain (main – subdomain)	Thematic category	Code	Description	Example quote	Strategy
				others to get this because...”	
Opportunity – social<br><br>Motivation – reflective	9. Community impact – cultural fit	9.3- CULTURAL_ APPROPRIATENESS	Comments on how well the calendar fits local culture, beliefs, and practices (e.g., alignment with traditions or contradictions).	“The calendar is similar to our traditional practices” or “Some advice contradicts what our community believe”	Deductive
Motivation – automatic<br><br>Capability – physical/psychological	10. MMS experience – sensory	10.1- ORGANOLEPTIC	Descriptions of MMS taste, smell, colour, or swallowability and how these sensory aspects affect willingness to take it.	“The smell of MMS is better than IFA”	Inductive
Motivation – automatic;<br><br>Capability – physical	10. MMS experience – side effects	10.2- PERCEIVED_ SIDE_ EFFECTS	Reports of perceived side effects associated with MMS (e.g., nausea, vomiting, constipation) and their impact on use.	“MMS made me feel nausea after taking it”	Inductive
Motivation – automatic	10. MMS experience – benefits	10.3- PERCEIVED_ BENEFITS	Perceived benefits after taking MMS (e.g., sleeping better, eating better, feeling stronger) that may reinforce adherence.	“After taking MMS I could sleep much better”	Inductive

COM-B domain (main – subdomain)	Thematic category	Code	Description	Example quote	Strategy
Capability – physical					
Motivation – reflective<br><br>Capability – physical	10. MMS experience – refusal	10.4- REFUSAL	Explicit refusal or inability to take MMS (e.g., stopping completely, rejecting the supplement).	“I couldn’t take MMS”	Inductive
