# Supplementary material for: Exploring Behavioral Interventions to Enhance Adherence to Multiple Micronutrient Supplementation Among Pregnant Women in Cambodia: A Mixed-Methods Study
Source: Nutrients. 2026 Feb 10;18(4):583. doi: 10.3390/nu18040583 (PMC12943067; doi:10.3390/nu18040583)
Supplement: Supplementary file 1 [file nutrients-18-00583-s001.zip › Supplementary Materials/Codebook MMS videos .pdf]

Summary COM-B–Informed Codebook for MMS Videos

COM-B domain (main – subdomain)	Thematic category	Code	Description	Example quote	Strategy
Motivation – automatic	1. Acceptability & relevance – overall impressions	1.1A-OI-POSITIVE	Positive feelings or experiences related to watching the MMS videos (e.g., feeling encouraged, reassured, or supported).	"The videos helped me to keep taking MMS daily"	Deductive
Motivation – automatic	1. Acceptability & relevance – overall impressions	1.1B-OI-NEGATIVE	Negative feelings or experiences related to the MMS videos (e.g., feeling worried, stressed, or burdened after watching).	"The videos made me feel more stressed about my pregnancy"	Deductive
Motivation – reflective	1. Acceptability & relevance – overall impressions	1.1C-OI-NEUTRAL	Neutral or mixed responses to the videos (e.g., finding them acceptable but not especially useful or impactful).	"The videos were ok, but didn't make much difference for me"	Deductive
Motivation – reflective	1. Acceptability & relevance – relevance of content	1.2A-RC-IMPORTANT	Information seen as important or serious, even if not directly linked to her own current experience or questions.	"They told us to abstain from drinking alcohol, smoking..."	Deductive

COM-B domain (main – subdomain)	Thematic category	Code	Description	Example quote	Strategy
Motivation – reflective	1. Acceptability & relevance – relevance of content	1.2B-RC-PERSONAL EXPERIENCE	Information perceived as relevant because it matches the woman's own pregnancy experiences or symptoms.	"The video showed morning sickness and I also experienced that too"	Deductive
Motivation – reflective	1. Acceptability & relevance – relevance of content	1.2C-RC-ANSWER QUESTIONS	Information perceived as helpful because it directly answered questions or doubts the woman had about MMS or pregnancy.	"The video answered my question whether to keep taking MMS after delivery"	Deductive
Motivation – reflective	1. Acceptability & relevance – relevance of content	1.2D-RC-NOT RELEVANT	Information perceived as not relevant to her needs (e.g., already known, not applicable, or unrelated to her situation).	"The information in the videos was not relevant for me as I already knew all of these topics"	Deductive
Capability – psychological	1. Acceptability & relevance – credibility of source	1.3A-CS-GENERAL	General expressions of trust in the videos without specifying a reason (e.g., trusting the information "100%").	"I trust it 100%"	Inductive
Capability – psychological	1. Acceptability & relevance – credibility of source	1.3B-CS-MIDWIFE	Trust is attributed to the presence or role of the midwife in the video (e.g., believing information because it is delivered by a health professional).	"I trusted the video because a midwife was sharing the information"	Deductive

COM-B domain (main – subdomain)	Thematic category	Code	Description	Example quote	Strategy
Capability – psychological	1. Acceptability & relevance – credibility of source	1.3C-CS-SAME AS ANC	Trust is attributed to consistency between video messages and information given at ANC visits.	“At my ANC visit they also talk about this information, so it makes me trust the video”	Deductive
Capability – psychological	1. Acceptability & relevance – credibility of source	1.3D-CS-EXPERIENCE	Trust is attributed to the alignment between video content and the woman’s own experiences (e.g., similar symptoms or situations).	“I experienced the same symptoms as the women in the video, so I trusted it"	Deductive
Capability – psychological	1. Acceptability & relevance – credibility of source	1.3E-CS-MORE EVIDENCE	Expressions of needing more evidence, time, or outcomes (e.g., baby’s health) before fully trusting the videos.	“I want to know whether my baby will be born healthy first”	Inductive
Capability – psychological	1. Acceptability & relevance – credibility of source	1.3F-CS-NOT CREDIBLE	Elements that reduced credibility or trust (e.g., scenes perceived as unrealistic or staged).	“When the midwife called the participant, I didn’t trust that was real”	Deductive
Capability – psychological	2. Usefulness for information & motivation – clarity	2.1A-CI-CLEAR	Participants found the information clear, simple, and easy to follow.	“The information was clear and easy to follow”	Deductive
Capability – psychological	2. Usefulness for information &	2.1B-CI-CONFUSING	Participant found parts of the information confusing, difficult to	“I had to rewatch the video several times as I didn’t understand”	Deductive

COM-B domain (main – subdomain)	Thematic category	Code	Description	Example quote	Strategy
	motivation – clarity		understand, or requiring rewatching.		
Capability – psychological;<br><br>Opportunity – physical;<br><br>Motivation – automatic	2. Usefulness for information & motivation – motivational impact	2.2A-MI-SUPPLEM ENT	Participant reports that videos helped her to start, improve, or maintain consistency in taking MMS.	"The videos helped me be more consistent taking my supplement"	Deductive
Capability – psychological<br><br>Motivation – reflective	2. Usefulness for information & motivation – motivational impact	2.2B-MI-DAILY ACTIVITES	Participants report that videos led to changes in their daily routines or self-care practices (e.g., sleep, rest, physical activity).	"After watching the videos I followed better sleeping habit"	Deductive
Capability – psychological<br><br>Motivation – reflective	2. Usefulness for information & motivation – motivational impact	2.2C-MI-FOOD	Participant reports that videos led to changes in diet or food choices (e.g., reducing sugary drinks, improving dietary quality).	"I stopped drinking sweet drinks"	Deductive
Capability – psychological<br><br>Motivation – reflective	2. Usefulness for information & motivation – motivational impact	2.2D-MI-NO CHANGE	Participant reports no behavioral change from the videos (e.g., behaviors already changed earlier due to ANC,	"The videos didn't make a difference in my habits" / "I already changed my eating	Inductive

COM-B domain (main – subdomain)	Thematic category	Code	Description	Example quote	Strategy
			calendar, or existing knowledge).	habits after reading the calendar"	
Capability – psychological<br>Opportunity – physical<br><br>Motivation – automatic	2. Usefulness for information & motivation – supplement reminder	2.3A-SR-BENEFITS	Reminder driven by explanations of MMS benefits for mother and/or baby, prompting women to stay consistent.	"The video told me again that MMS will help my baby so it reminded me to be consistent"	Deductive
Capability – psychological<br><br>Opportunity – physical<br><br>Motivation – automatic	2. Usefulness for information & motivation – supplement reminder	2.3B-SR-SICKNESS	Reminder to keep taking MMS despite morning sickness or other discomforts.	"Even with bloating I should still take MMS, I remember that when I am in pain"	Deductive
Capability – psychological;<br><br>Opportunity – physical<br><br>Motivation – automatic	2. Usefulness for information & motivation – supplement reminder	2.3C-SR-OTHER	Other visuals or messages (e.g., placement by the bed, routine timing) that reminded women about MMS.	"The placement of MMS next to bed and taking it daily at the same time"	Deductive

COM-B domain (main – subdomain)	Thematic category	Code	Description	Example quote	Strategy
Capability – psychological<br><br>Opportunity – physical<br><br>Motivation – automatic	2. Usefulness for information & motivation – calls to action	2.4A-CA-HEALTH CENTER	Identification of calls to action about when to go to the health center (e.g., missed menstruation, danger signs, first ANC visit).	"We should come to health center after missing menstruation to get supplement" or "Come to health center if experiencing danger signs"	Deductive
Capability – psychological<br><br>Opportunity – physical<br><br>Motivation – automatic	2. Usefulness for information & motivation – calls to action	2.4B-CA-HEALTHY PREGNANCY	Calls to action to follow healthy pregnancy practices (e.g., eating more vegetables, resting, avoiding harmful behaviours).	"We should eat more vegetables"	Deductive
Capability – psychological<br><br>Opportunity – physical<br><br>Motivation – automatic	2. Usefulness for information & motivation – calls to action	2.4C-CA-OTHER	Other calls to action presented in the videos (e.g., finishing MMS after delivery, continuing despite side effects).	"Finish all MMS even if after delivery" or "Continue taking supplement even if experiencing side effects"	Deductive

COM-B domain (main – subdomain)	Thematic category	Code	Description	Example quote	Strategy
Capability – psychological<br><br>Motivation – reflective	3. Improvements & preferences – content	3.1-CS (CONTENT_SUGGESTIONS)	Any suggestions for improving, adding, or removing information or messages in the videos.	"The nutrition advice should be more specific"	Deductive
Capability – psychological<br><br>Motivation – reflective	3. Improvements & preferences – content	3.1A-CS-ADD	Suggestions to add more information (e.g., additional pregnancy problems, specific advice, more examples).	"Include more information about managing common pregnancy problems"	Deductive
Capability – psychological<br><br>Motivation – reflective	3. Improvements & preferences – content	3.1B-CS-REMOVE	Suggestions to remove or reduce some content (e.g., messages perceived as repetitive or no longer needed).	"We are already taking MMS we don't need to be reminded to come to health center to get it"	Deductive
Capability – psychological<br><br>Motivation – reflective	3. Improvements & preferences – delivery style	3.2A-DS-LIKE	Participant liked the current delivery style with actors/role-play.	"I liked that the video had real people"	Deductive
Capability – psychological<br><br>Motivation – reflective	3. Improvements & preferences – delivery style	3.2B-DS-CHANGE CARTOONOR	Participant prefers a cartoon/animation format instead of actors.	"I wish the videos were animated"	Deductive

COM-B domain (main – subdomain)	Thematic category	Code	Description	Example quote	Strategy
		ANIMATIO N			
Capability – psychological<br><br>Motivation – reflective	3. Improvements & preferences – delivery style	3.2C-DS-OTHER	Participant suggests another style (e.g., real-life testimonials, different narrative approach).	"I wish the video was a testimonial of someone's experience taking MMS"	Deductive
Capability – psychological<br><br>Motivation – reflective	3. Improvements & preferences – dislikes/discomfo rt	3.3A-DD-ACTING	Dislike or discomfort with how characters acted, spoke, or interacted.	"I didn't like the tone the husband talked to the pregnant woman"	Deductive
Capability – psychological<br><br>Motivation – reflective	3. Improvements & preferences – dislikes/discomfo rt	3.3B-DD-INFORMAT ION	Dislike or discomfort about specific information shared in the video.	"I didn't like what they said about …"	Deductive
Capability – psychological<br><br>Motivation – reflective	3. Improvements & preferences – dislikes/discomfo rt	3.3C-DD-IMAGE	Dislike or discomfort regarding particular images, scenes, or visual depictions.	"I didn't like seeing the woman taking the supplement in her bed"	Deductive

COM-B domain (main – subdomain)	Thematic category	Code	Description	Example quote	Strategy
Capability – psychological<br><br>Motivation – reflective	3. Improvements & preferences – dislikes/discomfort	3.3D-DD-OTHER	Other sources of discomfort or dislike (e.g., video length, pacing, background details).	“The video was too short”	Deductive
Capability – psychological<br><br>Opportunity – physical<br><br>Motivation – automatic	4. Comparing interventions – ranking	4.1-INTERVENTION COMPARISON	Participants’ ranking of the three interventions (calendar, family support, videos) in order of preference or impact.	“I ranked the interventions Calendar first, family support second, videos third”	Deductive
Capability – psychological<br><br>Opportunity – physical<br><br>Motivation – automatic	4. Comparing interventions – most influential	4.2-MI (MOST INFLUENTIAL)	Which intervention is viewed as most influential in helping develop or maintain a daily MMS routine.	“Family support helped me the most to develop my supplement routine”	Deductive
Opportunity – social; Motivation – reflective	4. Comparing interventions – community usefulness	4.3-CU (COMMUNITY USEFULNESS)	Perceptions of whether other pregnant women in the community would find any of the three interventions useful.	“Other pregnant women would find the calendar helpful for reminding them to take MMS”	Deductive

COM-B domain (main – subdomain)	Thematic category	Code	Description	Example quote	Strategy
Capability – psychological; Opportunity – physical; Motivation – reflective	4. Comparing interventions – least effective	4.4-LE (LEAST EFFECTIVE)	Which intervention is perceived as least effective in supporting MMS adherence or behaviour change, and why.	"The videos didn't help me as I already changed behavior from the calendar"	Deductive
Motivation – automatic<br><br>Capability – physical/psychological	5. MMS experience – sensory	5.1-ORGANOLEPTIC	Descriptions of MMS taste, smell, appearance, or swallowability, and how these sensory aspects influence willingness to take it.	"The smell of MMS is better than IFA"	Inductive
Motivation – automatic<br><br>Capability – physical	5. MMS experience – side effects	5.2-PERCEIVED SIDE EFFECTS	Reports of perceived side effects from MMS (e.g., vomiting, nausea, constipation) and their impact on adherence.	"MMS made me feel nausea after taking it"	Inductive
Motivation – automatic<br><br>Capability–physical/psychological	5. MMS experience – benefits	5.3-PERCEIVED BENEFITS	Perceived benefits of MMS (e.g., sleeping better, eating better, feeling stronger) that may reinforce use.	"After taking MMS I could sleep much better"	Inductive

COM-B domain (main – subdomain)	Thematic category	Code	Description	Example quote	Strategy
Motivation – reflective<br><br>Capability – physical	5. MMS experience – refusal	5.4- REFUSAL	Explicit refusal or inability to take MMS (e.g., stopping completely, rejecting the supplement).	"I couldn't take MMS"	Inductive
