# Supplementary material for: Exploring Behavioral Interventions to Enhance Adherence to Multiple Micronutrient Supplementation Among Pregnant Women in Cambodia: A Mixed-Methods Study
Source: Nutrients. 2026 Feb 10;18(4):583. doi: 10.3390/nu18040583 (PMC12943067; doi:10.3390/nu18040583)
Supplement: Supplementary file 1 [file nutrients-18-00583-s001.zip › Supplementary Materials/FGD Guide_Intervention Family Support.pdf]

Discussion Guide: Family Support During Pregnancy

Date of FGD	
Moderator	
Note taker	
Province	

Approximately 60 minutes

Introduction (5 minutes):

Welcome, everyone, and thank you for joining us. We appreciate the opportunity to meet and discuss your current experiences with prenatal multiple micronutrient supplements.

You are invited to answer all questions throughout this discussion. The information you share with us will remain confidential, and your name will not be revealed to anyone outside of our team.

There are no right or wrong answers to the questions we will ask, and it is OK if you disagree with someone or have a different view. It is very useful for us to know everyone's different perspectives, so please don't be shy about sharing them with us! We invite you to respond to the comments and answers provided by other participants, and you can talk between yourselves throughout this discussion. We do ask that only one person speaks at a time, so we can hear everyone's answers.

Do you have any questions for us before we begin?

To help us get to know one another, could everyone introduce themselves? If you would like, you can also add a fun fact about yourself.

[Go around in a circle, starting from the left, and have everyone introduce themselves.]

Acceptability and Relevance (15 minutes):

1. **Overall Impression**: How did you feel about family involvement (burden/reminder)? (Open-ended)
   - **Probe:** Did you receive support or reminders from your family members about your pregnancy?

2. **Comfort Level:** Who were you most/least comfortable with regarding reminders (husband, mother-in-law, etc.)? Why? (Probe for specific family members)

3. **Communication Style:** Tell me about the style your family members used to support you? Was it helpful? Was it nagging? Too repetitive?
   - **Probe:** Can you give us an example.

Benefits and Drawbacks (20 minutes):

4. Positive Impact: Did reminders help develop a routine or habit of taking the supplement? How did family support impact your physical and mental well-being?
   - Probe: What exactly about the reminders was useful, for example its ability to help establish a habit formation and broader well-being

5. Challenges Faced: Did family involvement feel nagging or create tension? (Probe for any negative experiences) (Remind participants it's ok if they didn't always find the family support and reminders to be helpful)
   - Probe: Can you share an example of a time when their involvement felt overwhelming or unhelpful? What was it about their behavior that made you feel that way?

6. Comparison: Did family support make a difference in your supplement routine compared to before? How has their encouragement or understanding helped you feel about taking your supplement?
   - Probe: Was there any difference in your supplement routine from before your family members came to the support session compared to afterwards (the last 3 weeks)? What was the difference?

7. Benefits vs. Drawbacks: Briefly summarize the pros and cons you've mentioned. Which weighed more heavily?

Family Support Received (10 minutes):

8. Family Involvement (5 min): Did family members participate actively? Did participation vary (who, why)?
   - Probe: What was the support you received from family members over the past 3 weeks? Who provided this support?

9. Communication Frequency (5 min): Was the reminder frequency excessive, helpful, or insufficient? Why?

Recommendations and Future Improvements (10 minutes):

10. Future Intervention Design: How could family support be improved (focus on topics shared with family members during the session)

    Probe: What topics would you want your family members to be more knowledgeable about?

11. Recommendations for Family Members: What advice would you give family members supporting someone during pregnancy (reminders/motivation)? (Focus on practical advice)
    a. Probe: What would you want family members to say to you to support you during your pregnancy?
       Probe: Let's do a role play of one participant being the pregnant woman and the other being a husband (or other family member). Have the family member say a reminder to the pregnant woman in the way that they would want to receive in a reminder.

Wrap-up (5 minutes):

Summarize key points and thank participants for their valuable insights. Encourage further questions or comments.
