# Supplementary material for: Exploring Behavioral Interventions to Enhance Adherence to Multiple Micronutrient Supplementation Among Pregnant Women in Cambodia: A Mixed-Methods Study
Source: Nutrients. 2026 Feb 10;18(4):583. doi: 10.3390/nu18040583 (PMC12943067; doi:10.3390/nu18040583)
Supplement: Supplementary file 1 [file nutrients-18-00583-s001.zip › Supplementary Materials/FGD Guide_Intervention Videos.pdf]

Discussion Guide: Videos

Total time: 75 Minutes

Introduction (5 minutes):

Welcome, everyone, and thank you for joining us today. Briefly explain the purpose of the discussion, which is to get feedback on the social media videos about how to have a healthy pregnancy and to support adherence to prenatal supplements. Mention that the social media videos were co-designed by a nurse/midwife and endorsed by the health department.

You are invited to answer all questions throughout this discussion. Before answering a question, we will ask that you say your name or your participant number to help us keep track of who said what. The information you share with us will remain confidential, and your name will not be revealed to anyone outside of our team.

There are no right or wrong answers to the questions we will ask, and it is OK if you disagree with someone or have a different view. It is very useful for us to know everyone's different perspectives, so please don't be shy about sharing them with us! We invite you to respond to the comments and answers provided by other participants, and you can talk between yourselves throughout this discussion. We do ask that only one person speaks at a time, so we can hear everyone's answers.

Do you have any questions for us before we begin?

Overall Acceptability and Relevance (15 minutes):

1. **Overall Impression:** Did you find the videos sent by Helen Keller Staff to be informative and helpful during your pregnancy? Why or why not? (Open-ended)

2. **Relevance of Content:** Did the videos cover relevant and important topics to you during your pregnancy? Were there any crucial topics missing? (Focus on content relevance)

3. **Credibility of Source:** How credible and trustworthy did you find the videos? Why?
    - Probe: What about the videos make you trust or not trust the information in the videos?
    - Probe: Did having the midwife share the information in the videos influence whether you trust the videos?

Usefulness for Information and Motivation (20 minutes):

4. **Clarity of Information:** Was the information presented in the videos clear and easy to understand? Why or why not? (Focus on information quality)

5. **Motivational Impact:** Did the messages in the videos motivate you to focus on having a healthy pregnancy experience? How so?

    Probe: Did watching the videos influence you to change your habits during your pregnancy?

6. **Prenatal Supplement Reminder:** Did the videos effectively remind you about the importance of taking MMS? Why or why not? (Focus on effectiveness of reminder for MMS)

7. **Call to Action (5 min):** Did the videos provide clear calls to action, such as scheduling ANC visits or seeking further information? (Focus on clarity of action steps)

Improvements and Preferences (20 minutes):

8. **Content Suggestions:** Are there any specific topics you would like added or changed in video 1? Why or why not? What about video 2? Video 3? (Focus on desired content changes)

9. **Delivery Style:** Did you find the delivery style to be engaging and informative? Why or why not? Would you have preferred a different format (e.g., animation, cartoons, or testimonies)? (Focus on delivery style preferences)

10. **Unanswered Questions:** Did the videos raise any questions or concerns that were not addressed? (Focus on gaps in information)

11. **Discomfort or Dislike:** Was there anything about the videos that you found uncomfortable, confusing, or disliked? (Focus on negative feedback)

Comparing Interventions: Family Support, Wall Calendar, and Social Media Videos and Posts (15 minutes):

12. **Intervention Comparison (10 min):** We previously discussed family support, wall calendars, and social media videos and posts for promoting healthy pregnancy practices. Considering all three interventions, which one did you find the best to influence your behavior to take MMS daily? Why?

    Probe: Consider the information provided, motivation, social support, and personal preferences.

13. **Ranking Interventions:** [Provide a form for the ranking] If you could rank the three interventions from 1 (most effective) to 3 (least effective), what would your ranking be? What made you choose to put them in this order?

Wrap-up (5 minutes):

Summarize key points and thank participants for their valuable insights. Encourage further questions or comments.
