# Supplementary material for: Exploring Behavioral Interventions to Enhance Adherence to Multiple Micronutrient Supplementation Among Pregnant Women in Cambodia: A Mixed-Methods Study
Source: Nutrients. 2026 Feb 10;18(4):583. doi: 10.3390/nu18040583 (PMC12943067; doi:10.3390/nu18040583)
Supplement: Supplementary file 1 [file nutrients-18-00583-s001.zip › Supplementary Materials/FGD Guide_Intervention Wall Calendar.pdf]

Discussion Guide: Pregnancy Wall Calendar Intervention

Date of FGD	
Moderator	
Note taker	
Province	

Introduction (5 minutes):

Welcome, everyone, and thank you for joining us today. We want to learn your thoughts on the wall calendar and suggestions for improving it.

You are invited to answer all questions throughout this discussion. Before answering a question, we will ask that you say your name or your participant number to help us keep track of who said what. The information you share with us will remain confidential, and your name will not be revealed to anyone outside of our team.

There are no right or wrong answers to the questions we will ask, and it is OK if you disagree with someone or have a different view. It is very useful for us to know everyone's different perspectives, so please don't be shy about sharing them with us! We invite you to respond to the comments and answers provided by other participants, and you can talk between yourselves throughout this discussion. We do ask that only one person speaks at a time, so we can hear everyone's answers.

Do you have any questions for us before we begin?

Relevance and Acceptability (15 minutes):

1. **Overall Impression:** What were your overall impressions of the wall calendar?
   Probe: Did you use the calendar?

2. **Content Relevance:** What are your thoughts on the topics covered in the calendar?
   Probe: Were the topics covered in the calendar relevant and informative for your needs? Why or why not?
   Probe: Were there any topics missing that you think should be added?

3. **Visual Appeal:** What are your thoughts on the design and visual appeal of the calendar?
   Probe: What do you think about the colors? What about the layout? Did you find the calendar visually appealing and easy to use? Would you have preferred a different format? (Focus on visual design and format)

Usefulness as a Reminder (20 minutes):

4. **Frequency of Use:** How often did you refer to the calendar throughout your pregnancy? (Focus on frequency)
   Probe: Did you refer to it twice a day? Once a day? Once a week?

5. **ANC Checkup Reminders:** Were the reminders in the calendar about attending your ANC visits helpful to motivate you to attend your ANC checkups? How?
   Probe: Why or why not?

6. **Prenatal Vitamin Reminders:** Did the calendar act as a helpful reminder to take MMS? (Focus on reminder effectiveness for MMS).
   Probe: Why or why not?

7. **Visual Cues for Reminders:** Did the calendar's design or specific visuals effectively remind you about ANC checkups or taking your vitamins? How? (Focus on visual cues as reminders)

Motivational Messages and Information (20 minutes):

8. **Motivational Impact:** Did the calendar's messages and information motivate you to have a positive pregnancy experience? How? (Focus on overall motivational effect)

9. **Information Clarity:** Was the information in the calendar clear, easy to understand, and medically accurate? (Focus on information understanding and clarity)
   Probe: Was the information presented in trimester 1 clear and easy to understand? What about trimester 2? Trimester 3?

10. **Actionable Tips:** Did the calendar provide practical tips you could easily implement in your daily routine (e.g., healthy eating, exercise)? How?
    Probe: Why or why not?

11. **Comparison to Other Sources:** Did the calendar add value for you compared to other information that you already access (doctor, family members, online resources?) Why or why not?
    Probe: Would you buy the calendar for 4,000 Riels?

Wrap-up (5 minutes):

12. **Additional information:** Is there anything else you would like to share about your experience with the calendar?

Summarize key points and thank participants for their valuable insights. Encourage further questions or comments.
