# Supplementary material for: Exploring Behavioral Interventions to Enhance Adherence to Multiple Micronutrient Supplementation Among Pregnant Women in Cambodia: A Mixed-Methods Study
Source: Nutrients. 2026 Feb 10;18(4):583. doi: 10.3390/nu18040583 (PMC12943067; doi:10.3390/nu18040583)
Supplement: Supplementary file 1 [file nutrients-18-00583-s001.zip › Supplementary Materials/Tracking Calendar.pdf]

ម្ដាយមានសុខភាពល្អ កូនមានសុខភាពល្អ

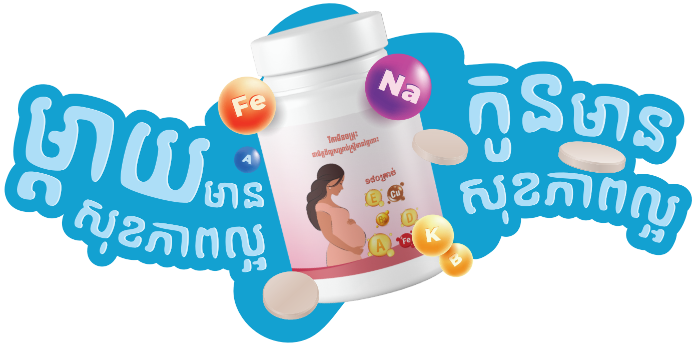

វាជាអាការៈធម្មតាជាទូទៅសម្រាប់ស្រ្តីមានផ្ទៃពោះ។
បន្តលេបថ្នាំមីក្រូសារជាតិចម្រុះ។

- ក្អួតចង្អោរ
- អស់កម្លាំងល្វិតល្វៃ
- ទល់លាមក
- ឈឺក្បាល

កុំភ្លេចលេបគ្រាប់ថ្នាំមីក្រូសារជាតិចម្រុះរបស់អ្នកជារៀងរាល់ថ្ងៃ!

គូស (✓) ជារៀងរាល់ថ្ងៃ
តាំងពីថ្ងៃដំបូង ដែលអ្នកចាប់ផ្តើម
លេបគ្រាប់ថ្នាំមីក្រូសារជាតិចម្រុះ។

1	2	3	4	5	6	7
8	9	10	11	12	13	14
15	16	17	18	19	20	21
22	23	24	25	26	27	28
29	30					

ថែរក្សាសុខភាពរបស់អ្នក
ក៏ដូចជាថែរក្សាសុខភាពកូនរបស់អ្នក

31	32	33	34	35	36	37
38	39	40	41	42	43	44
45	46	47	48	49	50	51
52	53	54	55	56	57	58
59	60					

អ្នកកំពុងកសាង
អនាគតដ៏ស្រស់បំព្រង

61	62	63	64	65	66	67
68	69	70	71	72	73	74
75	76	77	78	79	80	81
82	83	84	85	86	87	88
89	90					

សុខភាពរបស់អ្នក
ជាសុខភាពកូនរបស់អ្នក

91	92	93	94	95	96	97
98	99	100	101	102	103	104
105	106	107	108	109	110	111
112	113	114	115	116	117	118
119	120					

គ្រាប់ថ្នាំនីមួយៗ
ជួយឱ្យកូនអ្នកលូតលាស់បានល្អ

121	122	123	124	125	126	127
128	129	130	131	132	133	134
135	136	137	138	139	140	141
142	143	144	145	146	147	148
149	150					

ស៊ូៗ អ្នកម្ដាយគ្រប់រូប
អ្នកពិតជាអស្ចារ្យ!

151	152	153	154	155	156	157
158	159	160	161	162	163	164
165	166	167	168	169	170	171
172	173	174	175	176	177	178
179	180					

អ្នកធ្វើបានល្អណាស់!

សូមទៅជួបអ្នកផ្តល់សេវាថែទាំសុខភាព បើអ្នកមិនស្រួលខ្លួន ឬមានសញ្ញាគ្រោះថ្នាក់ដូចជា គ្រុនក្តៅ ឬធ្លាក់ឈាម។

THE CHURCH OF JESUS CHRIST OF LATTER-DAY SAINTS
Transforming Lives Through Nutrition
HELEN KELLER INTL | vitamin angels | iDE

ខែ 1-3

គួរធ្វើ៖

- លេបវីតាមីនរបស់អ្នក ជារៀងរាល់ថ្ងៃ
- សម្រាកនៅពេលដែលអ្នកមានអារម្មណ៍ថា អស់កម្លាំង ល្ហិតល្ហៃ
- ញ៉ាំអាហារតិចៗ តែញឹកញាប់ ៤ ដង ដើម្បីជួយកាត់បន្ថយ អាការៈចាញ់កូននៅពេលព្រឹក

មិនគួរធ្វើ៖

- កុំផឹកស្រា កុំជក់បារី កុំសេពគ្រឿងញៀន និងប្រើថ្នាំបុរាណ
- កុំបរិភោគ បន្លែ ផ្លែឈើដែលលាង សម្អាតមិនបានល្អ
- កុំផឹកកាហ្វេតែ និង សូដា ដែលមានជាតិកាហ្វេអ៊ីន ច្រើនលើសលុប

ខែ 4-6

គួរធ្វើ៖

- ជ្រើសរើសបរិភោគបន្លែ ផ្លែឈើ និងបរិភោគសាច់សុទ្ធ អត់មានខ្លាញ់
- ហាត់ប្រាណស្រាលៗ ដូចជាការដើរ
- លាងដៃឱ្យបានញឹកញាប់ និងលាងសម្អាតបន្លែ ផ្លែឈើ ឱ្យបានល្អ

មិនគួរធ្វើ៖

- កុំញ៉ាំសាច់ ស៊ុត ឬក៏ ត្រីដែលមិនបាន ចម្អិនឆ្អិនល្អ
- កុំញ៉ាំអាហារសម្រន់ ដែលមានជាតិស្ករខ្ពស់
- កុំភ្លេចលេបវីតាមីនសម្រាប់ ស្ត្រីមានផ្ទៃពោះ ជារៀងរាល់ថ្ងៃ

ខែ 7 -9

គួរធ្វើ៖

- ទៅពិនិត្យផ្ទៃពោះ ឱ្យបានទៀងទាត់
- ចែករំលែកពីអារម្មណ៍ របស់អ្នក ជាមួយក្រុម គ្រួសារ និង មិត្តភក្តិ
- ដើរយឺតៗ ឬ ពត់ខ្លួនយឺតៗ

មិនគួរធ្វើ៖

- កុំលើករបស់ធ្ងន់ ស្វែងរកជំនួយពីអ្នកក្បែរខ្លួន
- កុំឱ្យរអិលដួល
- កុំលោត ឬ ដាកលឿនពេក
