# Supplementary figures and images for: Exploring Behavioral Interventions to Enhance Adherence to Multiple Micronutrient Supplementation Among Pregnant Women in Cambodia: A Mixed-Methods Study
Source: Nutrients. 2026 Feb 10;18(4):583. doi: 10.3390/nu18040583 (PMC12943067; doi:10.3390/nu18040583)

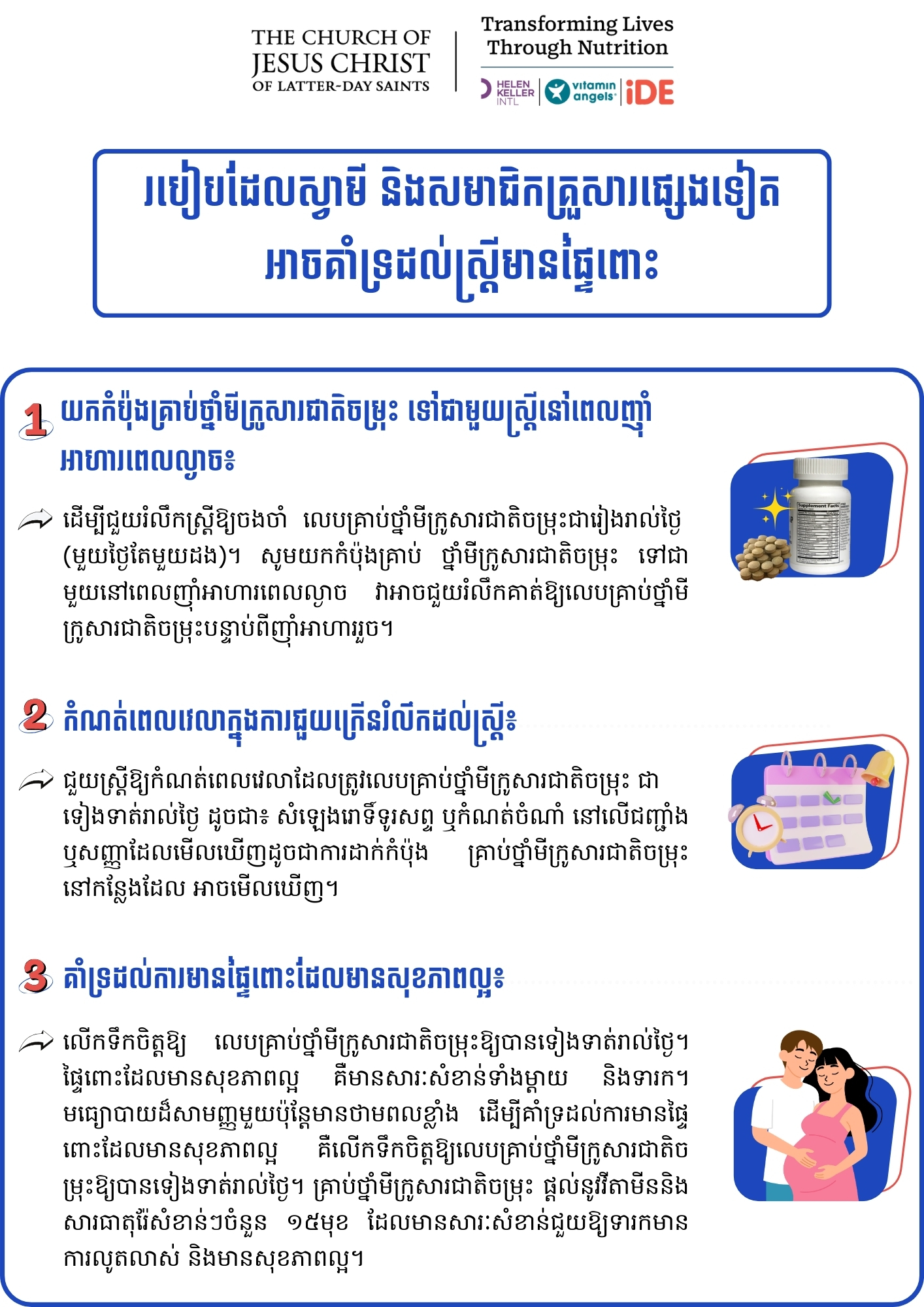

Supplement: Supplementary file 1 [file nutrients-18-00583-s001.zip › Supplementary Materials/Family Support Information Session Handout.jpg]
